# GR24, A Synthetic Strigolactone Analog, and Light Affect the Organization of Cortical Microtubules in Arabidopsis Hypocotyl Cells

**DOI:** 10.3389/fpls.2021.675981

**Published:** 2021-07-07

**Authors:** Yuliya Krasylenko, George Komis, Sofiia Hlynska, Tereza Vavrdová, Miroslav Ovečka, Tomáš Pospíšil, Jozef Šamaj

**Affiliations:** ^1^Department of Cell Biology, Centre of the Region Haná for Biotechnological and Agricultural Research, Faculty of Science, Palacký University Olomouc, Olomouc, Czechia; ^2^Department of Chemical Biology and Genetics, Centre of the Region Haná for Biotechnological and Agricultural Research, Faculty of Science, Palacký University Olomouc, Olomouc, Czechia

**Keywords:** microtubule organization, microtubule dynamics, kymographs, light, GR24, TIS108, *max2-1* mutant, Arabidopsis hypocotyl

## Abstract

Strigolactones are plant hormones regulating cytoskeleton-mediated developmental events in roots, such as lateral root formation and elongation of root hairs and hypocotyls. The latter process was addressed herein by the exogenous application of a synthetic strigolactone, GR24, and an inhibitor of strigolactone biosynthesis, TIS108, on hypocotyls of wild-type Arabidopsis and a strigolactone signaling mutant *max2-1 (more axillary growth 2-1)*. Owing to the interdependence between light and strigolactone signaling, the present work was extended to seedlings grown under a standard light/dark regime, or under continuous darkness. Given the essential role of the cortical microtubules in cell elongation, their organization and dynamics were characterized under the conditions of altered strigolactone signaling using fluorescence microscopy methods with different spatiotemporal capacities, such as confocal laser scanning microscopy (CLSM) and structured illumination microscopy (SIM). It was found that GR24-dependent inhibition of hypocotyl elongation correlated with changes in cortical microtubule organization and dynamics, observed in living wild-type and *max2-1* seedlings stably expressing genetically encoded fluorescent molecular markers for microtubules. Quantitative assessment of microscopic datasets revealed that chemical and/or genetic manipulation of strigolactone signaling affected microtubule remodeling, especially under light conditions. The application of GR24 in dark conditions partially alleviated cytoskeletal rearrangement, suggesting a new mechanistic connection between cytoskeletal behavior and the light-dependence of strigolactone signaling.

## Introduction

Following germination in the soil, the developing seedling grows in a manner defined by the surrounding physical conditions. Growth patterns, in this case, are related to the underground quality of light (Sheerin and Hiltbrunner, 2017; Yu and Huang, 2017), the mechanical impedance of the soil (e.g., Zhong et al., 2014; Yao et al., 2017), gravity (Zhu et al., 2019), and other factors. Such physical stimuli are integrated into whole plant responses of both the root and the aerial parts of the seedling by production, transport, and differential action of various plant hormones. These hormones, including ethylene, auxin, cytokinin, and others, may have opposing or additive roles during the early stages of seedling growth. For example, ethylene has inhibitory effects in dark conditions, and its action is canceled after the hypocotyl emerges from the soil and becomes exposed to ambient light (Yu and Huang, 2017). Meanwhile, auxin promotes hypocotyl elongation in light conditions, but has rudimentary effects in the dark (Jensen et al., 1998), while cytokinins establish an acropetal root gradient underlying the developmental zonation of the root (Montesinos et al., 2020).

Growth effects of plant hormones have been frequently correlated to their action on directional cell growth (Wang et al., 2020a) and as such, hormone signaling has been associated with inducible changes in cortical microtubule organization and dynamics. Since cortical microtubules play a role in cellulose deposition in the overlying cell wall, hormones promoting cell elongation trigger reorganization of cortical microtubules to transverse arrays, while hormones inhibiting cell elongation or promoting radial cell expansion induce either longitudinal or random cortical microtubule configurations (Chen et al., 2016; Wang et al., 2020c).

During hypocotyl hook formation that is developed underground to protect the shoot apical meristem from the unfavorable soil conditions, hypocotyl grows preferentially at the dorsal side of the hook owing to the accumulation of auxin to the ventral side, where growth is inhibited (Abbas et al., 2013). Later, the apical hook opens, and the growth of the hypocotyl becomes symmetric throughout its entire circumference. During hook formation and establishment, as well as opening and elongation, hypocotyl growth patterns are preceded by appropriate cortical microtubule patterning related to hormone action (Baral et al., 2021). In this line, auxins and gibberellins promote the transverse orientation of cortical microtubules and potentiate cell elongation (e.g., Locascio et al., 2013; Elliott and Shaw, 2018). Ethylene inhibits etiolated hypocotyl elongation while promoting a longitudinal microtubule orientation (Ma et al., 2016).

Hormone-induced microtubule reorganization might be a direct consequence of signaling and transcriptional regulation of yet undiscovered microtubule regulators (e.g., Pan et al., 2020; True and Shaw, 2020). The relationship between microtubule organization and hormonal signaling is often reciprocal, since effectors of the microtubule cytoskeleton have been found to regulate the distribution of hormonal receptors or transporters (e.g., Halat et al., 2020; Deng et al., 2021), and in this way contribute to their differential distribution.

Strigolactones, the carotenoid-derived plant hormones and rhizosphere signaling molecules, were discovered in exogenous allelochemical responses as germination stimulants of Orobanchaceae root parasitic weeds (*Striga, Orobanche, Phelipanche*, and *Alectra* spp.) (Cook et al., 1966; Koltai, 2014). Strigolactones initiate arbuscular mycorrhizal symbiosis (Akiyama et al., 2005; Taulera et al., 2020), promote nodulation in the legume-rhizobium symbiosis interaction (Foo et al., 2014; van Zeijl et al., 2015; McAdam et al., 2017), and enhance plant resistance to drought, salt and osmotic stresses, and low soil phosphate and nitrate content (Yoneyama et al., 2007; Foo et al., 2013; Ha et al., 2014; Min et al., 2019). The physiological effects of strigolactones on the aboveground plant part include: (i) regulation of plant height (de Saint Germain et al., 2013; Wang et al., 2020b), (ii) control of shoot branching by modulating auxin transport (Kapulnik et al., 2011; Shinohara et al., 2013; Bennett et al., 2016), (iii) suppression of the preformed axillary bud outgrowth (Gomez-Roldan et al., 2008; Umehara et al., 2008; Domagalska and Leyser, 2011), (iv) increased expansion of leaves (Hu et al., 2019) and cotyledons (Tsuchiya et al., 2010), (v) rescue of the dark-induced elongation of rice mesocotyls (Hu et al., 2010; Sun et al., 2018), (vi) promotion of the secondary growth (Agusti et al., 2011), (vii) regulation of meristem cell number in the root (Koren et al., 2013), and (viii) stimulation of leaf senescence (Koltai, 2014; Ueda and Kusaba, 2015). Furthermore, the synthetic strigolactone GR24 acts synergistically with auxins and it is involved in Arabidopsis seed germination (Toh et al., 2012), potato tuber formation, the outgrowth of the axillary stolon buds, and aboveground potato shoot branching (Roumeliotis et al., 2012).

At the cellular level, strigolactones and karrikins, the highly active germination stimulants of dormant seeds present in smoke and related signaling molecules represented by KAR1 to KAR6 compounds with butenolide ring (Waters et al., 2014), are differentially perceived by two related receptors, and their signals interconnect at the F-box protein MORE AXILLARY GROWTH2 (MAX2) (Nelson et al., 2011; De Cuyper et al., 2017; Wang et al., 2020b). The specific receptor for strigolactones in Arabidopsis is α/β-hydrolase DWARF14 (D14), while subsequent signaling requires the SKP1-CULLIN-F-BOX (SCF) complex, which leads to proteasome-mediated degradation of target proteins as SUPPRESSOR OF MAX2-LIKE 6, 7, and 8 (SMXL6, SMXL7, and SMXL8) and their orthologs (Kumar et al., 2015b; Soundappan et al., 2015; Wang et al., 2015; Seto et al., 2019). SMXL proteins can repress the signal and degrade in response to treatment with synthetic strigolactone analog *rac*-GR24 (Jiang et al., 2013; Stanga et al., 2013). It was recently found that SMAX1/SMXL2 regulate primary root and root hair development downstream of α/β hydrolase KAI2-mediated signaling in Arabidopsis (Villaécija-Aguilar et al., 2019). Strigolactones also affect auxin efflux through PIN-FORMED (PIN) auxin transporters (Ruyter-Spira et al., 2011; Koltai, 2014; Zhang et al., 2020) and SERINE/THREONINE-PROTEIN PHOSPHATASE 5 (PAPP5) (Struk et al., 2021).

Given the above information regarding the role of strigolactones and karrikins in plant morphogenetic processes, which are regulated in a hormone-dependent manner by cytoskeleton (Blume et al., 2017), we studied herein the effects of strigolactone content modulators on microtubule organization and dynamics. Plant cytoskeleton is involved in many developmental processes regulated by plant hormones, e.g., the switch from cell division to cell expansion (Ruan and Wasteneys, 2014), elongation and differentiation (Ivakov and Persson, 2013; Ambrose and Wasteneys, 2014; Sampathkumar et al., 2014), in plant responses to salt (Shoji et al., 2006) and osmotic stress (Komis et al., 2002; Wang et al., 2010), as well as in the formation of arbuscular mycorrhiza (Ho-Plágaro et al., 2021).

Previously, it was reported that strigolactones affect the architecture and dynamics of actin filaments in Arabidopsis root cells (Pandya-Kumar et al., 2014). Thus, GR24 reduces actin filament bundling in a MAX2-dependent manner and, at the same time, enhances F-actin dynamics, affects endosome trafficking and PIN2 localization at the plasma membrane (Pandya-Kumar et al., 2014). Moreover, plant responses to low phosphorus conditions involve MAX2-dependent reduction of PIN2 and endosome trafficking, plasma membrane polarization, and increased actin filament bundling in epidermal root cells (Kumar et al., 2015a). However, there are no studies on the strigolactone regulation on plant microtubules so far. Concerning this cytoskeletal component, it is only known that MEB55 and ST362 strigolactone analogs compromise the integrity of the microtubule network in highly invasive breast cancer cell lines (Mayzlish-Gati et al., 2015), and TIT3 and TIT7 strigolactone analogs may interfere with the microtubules in hepatocellular carcinoma cells (Hasan et al., 2018). Therefore, to be coherent with the cytoskeleton studies of Pandya-Kumar et al. (2014), where solely strigolactone-insensitive *max2* mutant plants crossed with Arabidopsis lines carrying TALIN-GFP and fABD2-GFP markers for actin visualization after GR24 treatment were used, we performed complementary *in vivo* studies of cortical microtubule organization and dynamics using the same *max2-1* mutant but crossed with lines carrying GFP-MBD (Marc et al., 1998) and GFP-TUA6 (Ueda et al., 2003) markers.

Here, we report the effects of exogenously applied synthetic strigolactone *rac-*GR24, and the inhibitor of strigolactone biosynthesis TIS108, on the organization and dynamics of cortical microtubules in epidermal cells of light-exposed and etiolated hypocotyls of wild-type plants and strigolactone-insensitive Arabidopsis mutant *max2-1*. Our results suggest that GR24 affects plant microtubule organization and dynamics under light and that this can be alleviated by etiolation.

## Materials and Methods

### Plant Material and Growth Conditions

Wild-type *Arabidopsis thaliana* plants, ecotype Columbia-0 (Col-0), and the strigolactone-insensitive *A. thaliana max2-1* mutant (EMS mutant in Col-0-background; Stirnberg et al., 2002), kindly provided by Prof. Hinanit Koltai (Institute of Plant Sciences ARO, Volcani Center, Bet-Dagan, Israel), were used in this study. Microtubule organization and dynamics were recorded in seedlings stably expressing a *35S::GFP–MBD* [microtubule-binding domain (MBD) of mammalian non-neuronal MICROTUBULE ASSOCIATED PROTEIN4] (Marc et al., 1998). Mutant plants of *max2-1* were crossed with a Col-0 line carrying *35S::GFP-MBD* or *35S::GFP-TUA6* (Ueda et al., 2003) constructs to mark microtubules. However, the GFP-TUA6 line suffers from a very low signal-to-noise ratio and also exhibits aberrant aggregations of spot-like fluorescence or accumulations of diffuse cytoplasmic fluorescence that cannot be cleaned up by image processing. This makes it difficult to use this line for the studies of microtubule dynamics on the SIM platform, though some supportive results are presented in Supplementary Figures 5, 6). For microscopy studies, the F3 generation of the progenies was used. Homozygous *max2-1* seedlings with the above microtubule marker and *max2-1* aboveground part phenotype were selected according to fluorescence detection under an epifluorescence microscope.

Prior to germination, seeds were sterilized in 1% v/v sodium hypochlorite solution supplemented with 0.1% v/v Tween-20 for 10 min, short-spin vortexed, immersed to 70% v/v ethanol for 5 s, thoroughly rinsed by MilliQ water, and placed to 0.6% w/v agarose-solidified ½ Murashige and Skoog medium (½ MS; Duchefa, Netherlands) with 1% w/v sucrose with or without exogenous synthetic strigolactone and/or inhibitors of strigolactone biosynthesis.

### Chemical Treatment

Unless stated otherwise, all common chemicals were purchased from Sigma-Aldrich. The generic synthetic strigolactone analog *rac*-GR24 was synthesized according to Wigchert et al. (1999). This compound is comprised of two enantiomers: D14-perceived (+)-GR24 (GR24*5DS) and non-specific KAI2-recepted (–)-GR24 (GR24*ent-5DS); they stimulate both the strigolactone and the karrikin signaling pathways and induce different physiological responses in Arabidopsis (Scaffidi et al., 2014; De Cuyper et al., 2017).

A synthetic *rac*-GR24 (further GR24) was dissolved *ex tempore* in anhydrous acetone according to the recommendations of Halouzka et al. (2020) to prepare 10 mM stock solution and used at 3 μM and 25 μM final concentrations. Generally, the effects of a wide concentration range of GR24 have been addressed before, including as high a concentration as 100 μM (e.g., Wang et al., 2020b). The GR24 concentrations chosen herein have been previously used as well and 25 μM is the highest concentration within the linear range of hypocotyl dose responses to the growth regulator (Jia et al., 2014).

A triazole-type strigolactone biosynthesis inhibitor, TIS108 (Chiralix, Netherlands), an effective tool for regulating strigolactone production in Arabidopsis (Ito et al., 2013) with some limitations (Kawada et al., 2019, 2020), was dissolved in pure acetone prior to use to make a 10 mM stock solution further diluted to 3 μM and 10 μM final concentrations and added to agarose-solidified ½ MS medium. A 0.01% v/v acetone solution in water was used as a mock control for both GR24 and TIS108.

Petri dishes with seeds were stored at 4°C overnight to synchronize germination and then germinated at a vertical position in Phytotron at 22°C under long-day conditions [16 h light/8 h darkness, photosynthetic photon flux (PPF): 120 μmol m^–2^ s^–1^] for 4 or 7 days prior to imaging. For the etiolation experiment, Petri dishes were wrapped in aluminum foil after seeding, stratified at 4°C overnight, and germinated as such under the same environmental conditions.

### Hypocotyl Growth Analysis

Petri dishes with 4–7-day-old seedlings were placed in a flatbed scanner (Image Scanner III, Seiko Epson, Japan) and scanned at transmitted light mode to document and subsequently quantify hypocotyl length. For hypocotyl width measurements, seedlings were documented with differential interference contrast under a wide field microscope (Axio Imager M2, Carl Zeiss, Germany), equipped with a polarizer and a Wollaston prism at three distinct parts of the hypocotyl: the upper part (beneath the cotyledon petiole); the middle part (at the mid-plane of hypocotyl); and the lower part (at the border with the primary root).

For detailed morphological studies, 4- and 7-day-old seedlings were captured using Axio ZoomV16 Stereo Zoom system (Carl Zeiss, Germany) in bright field illumination (objective lenses PlanApo Z 1.5x, FWD = 30 mm). Measurements were done using the default Measure application of ImageJ (Schneider et al., 2012) by tracking hypocotyls with the segmented line tool after appropriate scale calibration using the Set Scale tool of the Analyze menu. Measurements of hypocotyl growth were also performed using the NeuronJ plugin^1^ (Meijering, 2010) for ImageJ^2^ and the data are presented in Supplementary Figure 1.

### Microscopy

For live imaging of microtubules, two different Zeiss microscopy platforms (Zeiss Microscopy, Germany) were used (Komis et al., 2014, 2015). For deciphering microtubule organization, GFP conjugated with MBD molecular marker was visualized by means of CLSM with the LSM710 system (Carl Zeiss, Germany) equipped with a 63× Plan-Apochromat oil-immersion objective (1.40 NA) under excitation 488 nm, emission 510–540 nm.

Microscopy platform enabling structured illumination microscopy (SIM) (ELYRA PS.1, Carl Zeiss, Germany) with 63×/1.40 NA Plan-Apochromat oil-immersion objective was used for the time-lapse observations of microtubule dynamics. For the excitation of GFP, a BP495–575/LP750 filter was used (Vavrdová et al., 2020). In addition, 4-day-old seedlings were mounted between a microscope slide and a coverslip in 30 μL of liquid MS medium spaced by double-sided sticky tape and narrow Parafilm stripes, and they were extra sealed using liquid petroleum jelly (nail polish) to form a chamber prior to imaging for sample stabilization. This prevented the dislocation of the plantlets during the liquid exchange and allowed the observation of the same area for 2 h. Seedlings were grown at solidified GR24/TIS108-containing media for 4 days. Organization and dynamics of microtubules were observed in epidermal cells of the median part of hypocotyls of light-exposed and etiolated Arabidopsis seedlings.

All preparations with the etiolated seedlings were done quickly in a dark room using dim red or green light to prevent disturbances of microtubules.

### Post-acquisition Image Processing

Raw SIM images were processed automatically by the respective add-on of the licensed Zen software (Black version; Carl Zeiss, Germany) coupled to the Elyra PS.1, according to standards thoroughly described before (Komis et al., 2014, 2015).

Kymographs of microtubule time series recordings were generated with the Kymograph add-on of the licensed Zen software (Blue version; Carl Zeiss, Germany), using the arrow tool to delineate individual or bundled microtubules of interest.

### Quantitative Analysis of Microtubule Organization

Microtubule organization was quantitatively addressed by assessing the extent of microtubule bundling as the skewness of fluorescence distribution of GFP-MBD-expressing cells. Skewness is automatically extrapolated by histogram analysis by means of Zen Blue software (Carl Zeiss, Germany) and it is defined as a deviation from a continuous distribution, which is symmetric around the mean value. When the dataset is skewed, the curve is either one-tailed or two-tailed with unequal tails and the program delivers a value corresponding to the co-efficient of skewness, which is derived as:

$$\begin{array}{c} Skewness=\frac{1}{N}\sum_{I=1}^{N}{\left(\frac{I_{N}-\bar{I}}{SD}\right)}^{3}, \end{array}$$

where *I*_*N*_ = any given value of fluorescence intensity; $\bar{I}$ = the mean intensity value; *N* = the size of the dataset (i.e., the number of fluorescence intensity values); *SD* = the standard deviation [adapted from Higaki et al. (2010)].

Skewness practically evaluates how much the fluorescence intensity of each given pixel in the image deviates from the mean value, or, in other words, how uniformly the sample is labeled. When the sample is uniformly labeled (i.e., when there is uniform background fluorescence or in our case when microtubules are uniformly distributed in the cortical cytoplasm), the skewness value is zero. When fluorescence distribution is non-uniform (i.e., when there are distinct dark and fluorescent spaces) and depending on how much non-uniform it is (i.e., dark and fluorescent structures are unevenly distributed), then it is considered to be skewed and has values different than zero. Evidently, when microtubules are bundled (i.e., when parallel microtubules are coming to a distance smaller than the resolution limit of the microscope), skewness increases, and it does so proportionally, according to the width of the bundle. In terms of fluorescence intensity, microtubule bundling is addressed here as the merging of at least two distinct microtubules and having an intensity value at least twice that of an apparently single microtubule (Burkart and Dixit, 2019).

The degree of cortical microtubule ordering was quantitatively assessed by measuring microtubule organization anisotropy, which is defined as the existence of a dominant microtubule orientation as compared to the main cell axis (Landrein and Hamant, 2013). Microtubule anisotropy was qualitatively demonstrated in maximum intensity projections of CLSM images of hypocotyl cells expressing GFP-MBD analyzed with the Cytospectre freeware (Kartasalo et al., 2015), which illustrates the angular distribution of microtubules in selected regions of interest (ROIs) plotted as circular graphs. The narrower this distribution becomes, the more prevalent is a given orientation of microtubule organization.

Quantitatively, the ordering of cortical microtubules was measured through the FibrilTool macro as described previously (Boudaoud et al., 2014). Briefly, the FibrilTool macro was applied on ROIs drawn using the Polygon tool of ImageJ delineating the circumference of fully visible cells. Care was taken, to avoid cell edges, where frequently the signal is saturated and would be falsely added to the result. Theoretically, the numerical result ranges between 0 (complete isotropy; i.e., uniform distribution of cortical microtubules with no prevalent orientation) to 1 (perfect anisotropy; i.e., biased arrangement of cortical microtubules to one orientation).

### Quantitative Assessment of Microtubule Dynamics

Kymographs from recordings of dynamic microtubules were used to extrapolate the following parameters of microtubule dynamics: growth and shrinkage rates, catastrophe and rescue frequencies. Kymograph analysis was done manually using the ImageJ angle measure tool after size calibration of kymographs. Angles were acquired in degrees and converted to radians in MS Excel (Microsoft, United States) prior to the calculation of tangential values. Briefly, the equations used were as follows:

For growth rate, the equation was: *G* = *tan φ* × *pixel size* × *fps*, where *tan φ* is the tangential of the growth slope, *pixel size* is in μm and *fps* is the frame rate of the acquisition (frames × s^–1^). The final output is converted to μm × min^–1^ by multiplying the original value with 60 s × min^–1^.

For shrinkage rate, the equation was: *S* = *tan θ* × *pixel size* × *fps*; *tan θ* is the tangential of the shrinkage slope, *pixel size* is in μm and *fps* is the frame rate of the acquisition (frames × s^–1^). The final output is converted to μm × min^–1^ by multiplying the original value with 60 s × min^–1^.

For catastrophe frequency the following equation was applied:

$$\begin{array}{c} f_{cat}=\frac{{\text{N}}_{cat}}{t_{growth}}, \end{array}$$

where *f*_*cat*_ is the catastrophe frequency, *N*_*cat*_ is the total number of catastrophe events, and Σ*t*_*growth*_ is the total time spent in growth, regarding all the growth events considered.

For rescue frequency the following equation was applied:

$$\begin{array}{c} f_{res}=\frac{{\text{N}}_{res}}{t_{shrinkage}}, \end{array}$$

where *f*_*res*_ is the rescue frequency, *N*_*res*_ is the total number of rescue events, and Σ*t*_*growth*_ is the total time spent in shrinkage, regarding all the shrinkage events considered.

All measures from individual microtubules (N) taken into consideration for kymograph analysis were done on SIM videos and circa 10 cells were analyzed for each treatment.

### Statistics

Statistical analysis of all datasets was performed in the software STATISTICA (version 13.4.0.14; Statsoft, United States). All datasets were first subjected to the Shapiro–Wilk *W* test and Levene’s tests to test the normality and homogeneity. Frequently, the datasets failed to pass these tests. On several representative datasets, the following tests were calculated: (i) one-way ANOVA; (ii) Welch’s ANOVA, both followed either by Tukey’s *post hoc* test corrected for unequal sample size or Scheffé’s *post hoc* test; and (iii) Kruskal–Wallis test. Based on the results of these preliminary analyses, and in agreement with previous reports (Liu, 2015), Welch’s ANOVA followed by Scheffé’s test was used in most cases as it exhibited higher stringency compared to other tests. Statistical significance was determined based on the calculated *p*-values, were for Welch’s ANOVA the probability level was 0.05 and for Scheffé’s test, the probability level was 0.01. For comparing two different experimental conditions (light/darkness and inhibitor treatment), two-way ANOVA followed by Scheffé’s test was used. In this case, the probability level set for Scheffé’s test was 0.001.

## Results

### GR24 Affects Hypocotyl Growth in Arabidopsis

**FIGURE 1:**
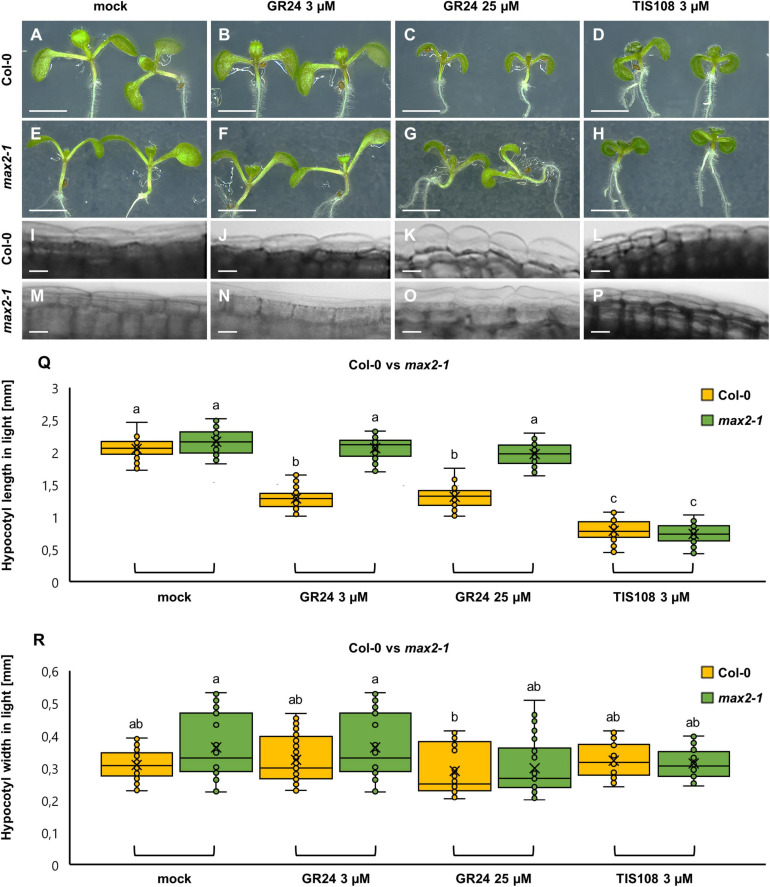
Hypocotyl development of light-grown seedlings of *Arabidopsis* Col-0 or the *max2-1* mutant in the presence or absence of the synthetic strigolactone GR24 (3 μM and 25 μM) or the biosynthetic inhibitor of strigolactone production TIS108 (3 μM). **(A–D)** Overview of hypocotyl of Col-0 seedlings treated with solvent alone (mock) **(A)**, 3 μM GR24 **(B)**, 25 μM GR24 **(C)**, and 3 μM TIS108 **(D)**. **(E–H)** Similar overview of hypocotyls of light-grown *max2-1* mutant seedlings in mock **(E)**, 3 μM GR24 **(F)**, 25 μM GR24 **(G)**, and 3 μM TIS108 **(H)**. **(I–L)** Magnified views of the middle part of hypocotyls of Col-0 treated with mock **(I)**, 3 μM GR24 **(J)**, 25 μM GR24 **(K)**, and 3 μM TIS108 **(L)** showing cell swelling in all treatments **(J–L)** compared to mock **(I)**. **(M–P)** A similar comparison was made of *max2-1* hypocotyl epidermal cells treated with mock **(M)**, 3 μM GR24 **(N)**, 25 μM GR24 **(O)**, and 3 μM TIS108 **(P)**. **(Q)** Quantitative assessment of Col-0 and *max2-1* hypocotyl length comparing pairwise mock treatment and treatments with 3 μM GR24, 25 μM GR24, and 3 μM TIS108 (*N* ≥ 59; two-way ANOVA was followed with Scheffé’s test; a statistical comparison is shown within groups sharing the same genotype; letters in the graph are shared by groups without statistically significant differences at the 0.001 probability level; results are in Supplementary Table 1). **(R)** Quantitative assessment of Col-0 and *max2-1* hypocotyl width comparing pairwise mock treatment and treatments with 3 μM GR24, 25 μM GR24, and 3 μM TIS108 (*N* ≥ 27; two-way ANOVA was followed with Scheffé’s test; a statistical comparison is shown within the groups sharing the same genotype; letters in the graph are shared by groups without statistically significant differences at the 0.001 probability level; results are in Supplementary Table 2). In all box plots, the average is presented by ×, median by the middle line, 1^st^ quartile by the bottom line, 3^rd^ quartile by the top line; the whiskers lie within the 1.5× interquartile range (defined from the 1^st^ to the 3^rd^ quartiles), while outliers are omitted. Scale bars: 5 mm **(A–H)**; 5 μm **(I–P)**.

Col-0 and *max2-1* mutant seedlings were germinated and cultivated on a medium containing different concentrations of GR24 or TIS108, either in the standard light/dark regime or just in the dark. Under the light/dark regime, GR24 was applied at two different concentrations (3 μM and 25 μM), stalled hypocotyl elongation (Figure 1). At 3 μM there was no visible radial expansion of hypocotyl epidermal cells (Figure 1J) and no apparent swelling of the hypocotyl (Figure 1R), but at 25 μM, GR24 promoted visible cell swelling and mild radial expansion of the hypocotyl compared to mock-treated Col-0 controls (Figures 1A,I cf. Figures 1B,C,J,K,R). On the other hand, treatment with 3 μM of TIS108 caused growth inhibition of the hypocotyl without radial epidermal cell swelling or lateral expansion of the hypocotyl (Figures 1D,L). In quantitative terms, the hypocotyl length of mock-treated Col-0 seedlings comprised 2.05 ± 0.145 mm (mean ± SD; Figure 1Q; *N* = 75; Supplementary Table 1). After treatment with 3 μM GR24, the hypocotyl length was significantly reduced to 1.28 ± 0.158 mm (mean ± SD; Figure 1Q; *p* = 0.0000; *N* = 60), while after treatment with 25 μM GR24 the hypocotyl length comprised 1.307 ± 0.178 (mean ± SD; Figure 1Q; *N* = 56), which was significantly different compared to mock-treated Col-0, but not different from the effect of 3 μM GR24 (*p* = 0.0000 and *p* = 0.9992, respectively). In turn, TIS108 treatment resulted in the most severe hypocotyl growth inhibition. In this case, hypocotyl length was measured to 0.783 ± 0.160 mm (mean ± SD; Figure 1Q; *N* = 61), being significantly different from all other conditions tested (*p* = 0.0000 as compared to both treatments with 3 μM and 25 μM GR24). Hypocotyl width was only slightly affected by any of the treatments used herein (Figure 1R). Briefly, the width of mock-treated Col-0 hypocotyls was 0.309 ± 0.04 mm (Figure 1R; *N* = 58; Supplementary Table 2), 0.325 ± 0.07 mm after treatment with 3 μM GR24 (Figure 1R; *N* = 89), 0.290 ± 0.08 mm after treatment with 25 μM GR24 (Figure 1R; *N* = 90), and 0.323 ± 0.05 mm after treatment with 3 μM TIS108 (Figure 1R; *N* = 54).

Subsequently, we characterized hypocotyl growth in light-exposed *max2-1* mutants. In such mock-treated mutants (Figure 1E) as well as after the treatment with both 3 μM (Figure 1F) and 25 μM GR24 (Figure 1G), the hypocotyl length was comparable to mock-treated Col-0 seedlings. In contrast, *max2-1* hypocotyls were affected by TIS108 treatment, appearing shorter than those of mock-treated *max2-1* seedlings (Figure 1H). In all treatments, the hypocotyl width of *max2-1* seedlings did not show any noticeable changes (Figures 1M–P). In quantitative terms, the hypocotyl length of mock-treated *max2-1* mutants was 2.16 ± 0.19 mm (mean ± SD; *N* = 78), 2.06 ± 0.17 mm after 3 μM GR24 (mean ± SD; *N* = 69), 1.98 ± 0.17 mm after 25 μM GR24 (mean ± SD; *N* = 80), and 0.74 ± 0.16 mm after 3 μM TIS108 (mean ± SD; *N* = 59) treatments. The hypocotyl length of mock-treated *max2-1* mutants was comparable to the hypocotyl length of Col-0 controls but it was significantly different at all GR24 treatments, as compared to the similarly treated Col-0 seedlings (*p* = 0.0000 after 3 μM GR24 and 25 μM GR24). Within the *max2-1* population, GR24 treatments did not affect hypocotyl length (Figure 1Q; *p* = 0.185 after mock treatment, *p* = 0.9999 after 3 μM GR24, and *p* = 0.2333 after 25 μM GR24 treatments) and only treatment with 3 μM TIS108 brought about its significant shortening (*p* = 0.0000 after 3 μM TIS108; Figure 1Q). In terms of hypocotyl width, no changes were discerned in *max2-1* seedlings (Figure 1R).

### GR24 Effects Are Modulated in Dark-Grown Seedlings

Since several reports of a synergy between exogenous application of strigolactones and the illumination conditions during seedling growth exist (e.g., Brewer et al., 2013; Jia et al., 2014), the experimental regime of the treatment of Col-0 and *max2-1* seedlings with two different concentrations of GR24 (3 μM and 25 μM) and with 3 μM TIS108 grown under persistent darkness was tested.

**FIGURE 2:**
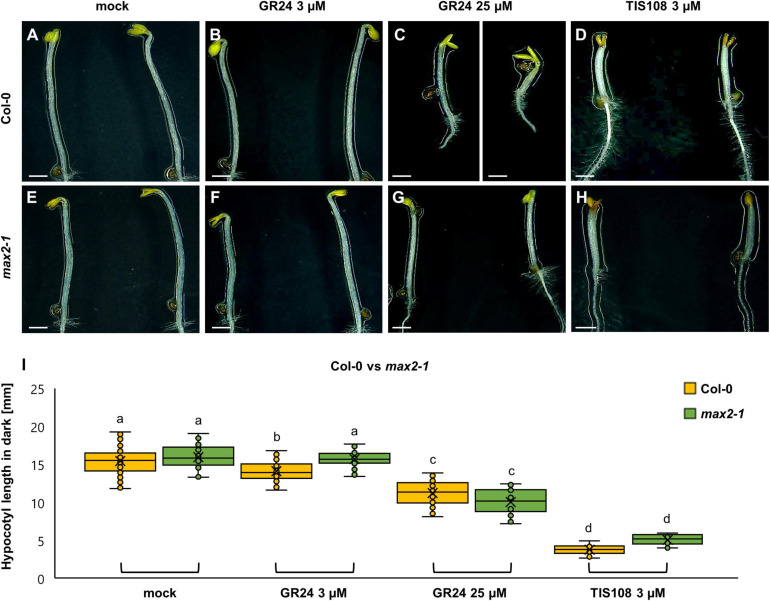
Hypocotyl development of dark-grown seedlings of *Arabidopsis* Col-0 or the *max2-1* mutant in the presence or absence of the synthetic strigolactone GR24 (3 μM and 25 μM) or the biosynthetic inhibitor of strigolactone production TIS108 (3 μM). **(A–D)** Overview of the etiolated hypocotyl of Col-0 seedlings treated with solvent alone (mock) **(A)**, 3 μM GR24 **(B)**, 25 μM GR24 **(C)**, and 3 μM TIS108 **(D)**. **(E–H)** Similar overview of hypocotyls of etiolated *max2-1* mutant seedlings in the presence of mock **(E)**, 3 μM GR24 **(F)**, 25 μM GR24 **(G)**, 3 μM of TIS108 **(H)**. **(I)** Quantitative assessment of etiolated Col-0 and *max2-1* hypocotyl length comparing pairwise mock treatment and treatments with 3 μM GR24, 25 μM GR24, and 3 μM TIS108 (*N* ≥ 22; two-way ANOVA was followed with Scheffé’s test; a statistical comparison is shown within the groups sharing the same genotype; letters in the graph are shared by groups without statistically significant differences at the 0.001 probability level; results are in Supplementary Table 3). In all box plots, the average is presented by ×, median by the middle line, 1^st^ quartile by the bottom line, 3^rd^ quartile by the top line; the whiskers lie within the 1.5× interquartile range (defined from the 1^st^ to the 3^rd^ quartiles), while outliers are omitted. Scale bars: 10 mm **(A–H)**.

As expected, hypocotyl length elongation of etiolated seedlings exceeds that of light-grown ones (Figure 2A). By combining visual documentation and quantitative analysis, it became evident that etiolated seedlings were mildly, if at all, responsive to treatments with 3 μM GR24 (Figures 2B,I), but more obviously after treatment with 25 μM GR24 (Figures 2C,I). Meanwhile, inhibition of etiolated hypocotyls in Col-0 was the most prominent after 3 μM TIS108 (Figures 2D,I). Thus, the length of mock-treated etiolated Col-0 seedlings was 15.45 ± 1.77 mm (mean ± SE; *N* = 78), 14.09 ± 1.22 mm after treatment with 3 μM GR24 (mean ± SE; *N* = 75), 11.23 ± 1.54 mm after 25 μM GR24 (mean ± SE; *N* = 77), and 2.55 ± 0.72 mm after 3 μM TIS108 (*N* = 13) treatments. Although it was not visible in all cases, GR24 caused a significant reduction of the etiolated hypocotyl length as compared to the mock treatment (Figure 2I; *p* = 0.0000 for 3 μM GR24; *p* = 0.0000 for 25 μM GR24; *p* = 0.0000 for 3 μM TIS108; Supplementary Table 3).

The same line of experiments was applied in the case of *max2-1* mutants (Figures 2E–H), which also proved to be prone to either the application of exogenous strigolactone, or to the metabolic inhibition of strigolactone biosynthesis. Hypocotyl length of 3 μM GR24-treated seedlings (Figures 2F,I) was 15.72 ± 1.12 mm (mean ± SD; *N* = 30), of 25 μM GR24-treated (Figures 2G,I) was 10.05 ± 1.66 mm (mean ± SD; *N* = 22), and of 3 μM TIS108-treated ones (Figures 2H,I) was 3.07 ± 0.66 mm (mean ± SD; *N* = 19), respectively, as compared to 15.95 ± 1.64 mm (mean ± SD; *N* = 23) of mock-treated *max2-1* seedlings (Figures 2E,I). The effect of most treatments on hypocotyl length of etiolated *max2-1* seedlings was deemed to be significant by comparison to the mock treatment, except for 3 μM GR24 (Figure 2I; *p* = 0.9998 for 3 μM GR24; and *p* = 0.0000 for both 25 μM GR24 and 3 μM TIS108).

To assess whether light modulates GR24 effects, we examined the extent of its influence on the percentage of hypocotyl reduction of either light- or dark-grown Col-0 or *max2-1* mutant seedlings by comparison to the mock treatment. Thus, in light exposed Col-0 seedlings treated with 3 μM GR24, hypocotyl length was reduced to 63.33 ± 9.68% in Col-0 (Supplementary Figure 2A) and to 93.01 ± 12.48% in etiolated seedlings (Supplementary Figure 2C) as compared to mock-treated seedlings. Similarly, the treatment with 25 μM GR24 caused the reduction of hypocotyl length up to 64.35 ± 9.63% of mock-treated seedlings under light exposure, but this reduction was less pronounced in dark (cf. 74.00 ± 13.69% of mock-treated seedlings). TIS108 treatment caused hypocotyl length reduction in light up to 38.55 ± 8.17% of mock-treated seedlings and an even more pronounced reduction in the darkness since the treated etiolated hypocotyls were 23.90 ± 4.61% of the mock-treated counterparts.

Regarding the light-exposed *max2-1* mutant, hypocotyl length of seedlings treated with 3 μM GR24 (Supplementary Figure 2B) was 96.65 ± 11.09% of mock-treated seedlings, of those treated with 25 μM GR24—91.88 ± 10.55%, and of those treated with 3 μM TIS108—34.72 ± 8.02%, respectively. Moreover, hypocotyl length of etiolated *max2-1* seedlings (Supplementary Figure 2D) after the treatment with 3 μM GR24 was 97.38 ± 6.92% of mock-treated seedlings, of those treated with 25 μM GR24—63.18 ± 6.39%, and of those treated with 3 μM TIS108—19.74 ± 3.19%, respectively.

These results revealed the extent of GR24 and TIS108 effects on hypocotyl elongation, showing that etiolated Col-0 seedlings are less prone to growth inhibition at 3 μM GR24 as compared to light-grown ones. However, they appear very responsive to 25 μM GR24, while at the same time they were also sensitive to TIS108 treatment. In addition, *max2-1* mutants were equally unresponsive to 3 μM GR24, however, both 25 μM GR24 and 3 μM TIS108 caused a much stronger inhibitory effect in etiolated seedlings. The above observations suggest that in the absence of light the effects of GR24 are modified.

### GR24 Affects Microtubule Organization in a Light-Dependent Manner

Inducible growth alterations following extrinsic stimulation as, e.g., with hormonal treatments, has been repeatedly shown to be preceded and supported by conditional rearrangements of cortical microtubules, which tend to organize parallel to each other in support of cell growth directionality (e.g., Lindeboom et al., 2013; True and Shaw, 2020). Such conditions favoring the parallel arrangement of cortical microtubules, can be documented by showing the patterns of their angular distribution and quantified by measuring the degree of anisotropy within the cortical array. Anisotropy is a measure of how well microtubules are organized, and not in which direction their organization occurs, i.e., it is affected by the numbers of microtubules in a prevalent orientation vs. the numbers of discordant microtubules.

**FIGURE 3:**
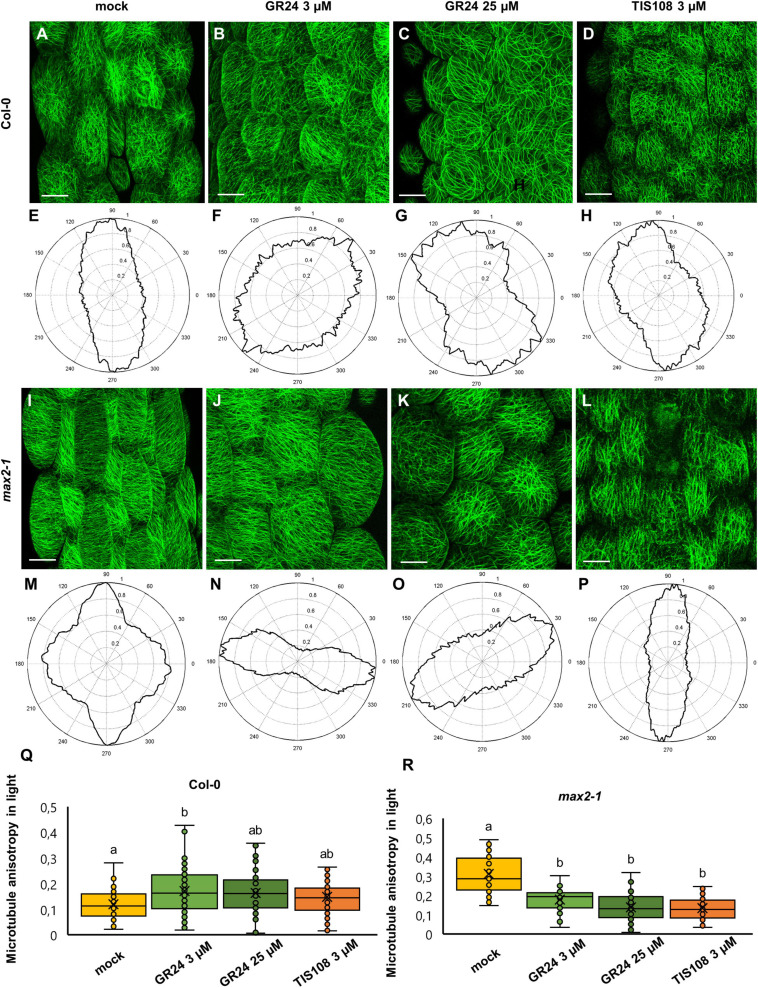
Assessment of microtubule organization in epidermal hypocotyl cells of light-grown seedlings of *Arabidopsis* Col-0 or the *max2-1* mutant expressing GFP-MBD marker in the presence or absence of the synthetic strigolactone GR24 (3 μM and 25 μM) or the biosynthetic inhibitor of strigolactone production TIS108 (3 μM). **(A–D)** Overview of hypocotyl of Col-0 seedlings treated with mock **(A)**, 3 μM GR24 **(B)**, 25 μM GR24 **(C)**, 3 μM TIS108 **(D)**. **(E–H)** Cytospectre graphs of cortical microtubule distribution, where panel **(E)** corresponds to panel **(A)**, panel **(F)** to panel **(B)**, panel **(G)** to panel **(C)**, and panel **(H)** to panel **(D)**. **(I–L)** Overview of hypocotyl of *max2-1* seedlings treated with mock **(I)**; 3 μM GR24 **(J)**, 25 μM GR24 **(K)**, 3 μM TIS108 **(L)**. **(M–P)** Cytospectre graphs of cortical microtubule distribution, where panel **(M)** corresponds to panel **(I)**, panel **(N)** to panel **(J)**, panel **(O)** to panel **(K)**, and panel **(P)** to panel **(L)**. **(Q)** Quantitative assessment of anisotropy of cortical microtubule organization in etiolated Col-0 after mock treatment and treatments with 3 μM GR24, 25 μM GR24, and 3 μM TIS108 (*N* ≥ 54; Welch’s ANOVA was followed with Scheffé’s test; a statistical comparison is shown within groups sharing the same genotype; letters in the graph are shared by groups without statistically significant differences at the 0.01 probability level; results are in Supplementary Table 4). **(R)** Quantitative assessment of anisotropy of cortical microtubule organization in etiolated *max2-1* after mock treatment and treatments with 3 μM GR24, 25 μM GR24, and 3 μM TIS108 (*N* ≥ 31; Welch’s ANOVA was followed with Scheffé’s test; a statistical comparison is shown within groups sharing the same genotype; letters in the graph are shared by groups without statistically significant differences at the 0.01 probability level; results are in Supplementary Table 5). In all box plots, the average is presented by ×, median by the middle line, 1^st^ quartile by the bottom line, 3^rd^ quartile by the top line; the whiskers lie within the 1.5× interquartile range (defined from the 1^st^ to the 3^rd^ quartiles) while outliers are omitted. Scale bars: 20 μm.

In light-grown, mock-treated Col-0 seedlings expressing a GFP-MBD microtubule marker, cortical microtubules in epidermal cells of the median part of Arabidopsis hypocotyls, exhibit a more or less random distribution with the predominantly longitudinal orientation relative to the cell longitudinal axis (Figures 3A,E) with the tendency of more disturbed organization after the treatment with 3 μM GR24 (Figures 3B,F) and the reorientation of the microtubule array after 25 μM GR24 (Figures 3C,G) and 3 μM TIS108 (Figures 3D,H) treatments as compared to mock (Figures 3A,E). In turn, *max2-1* mutants expressing the same microtubule marker, appeared to have more ordered cortical microtubules after treatments with 3 μM GR24 (Figures 3J,N), 25 μM GR24 (Figures 3K,O), and 3 μM TIS108 (Figures 3L,P), as compared to the treated Col-0 seedlings (Figures 3B–D). Generally, *max2-1* mutant group has less random microtubule arrays (Figures 3J–L) as compared to Col-0 (Figures 3B–D).

The qualitative observations mentioned above were quantitatively corroborated by measuring changes in the anisotropy of microtubule organization. In mock-treated Col-0 anisotropy was 0.12 ± 0.06 (Figure 3Q; mean ± SD; *N* = 54), after treatment with 3 μM GR24 it became 0.17 ± 0.08 (Figure 3Q; mean ± SD; *N* = 71), and 0.16 ± 0.08 (Figure 3Q; mean ± SD; *N* = 54) and 0.15 ± 0.07 (Figure 3Q; mean ± SD; *N* = 75) after 25 μM GR24 and 3 μM TIS108 treatments, respectively. In light-grown Col-0 seedlings, only 3 μM GR24 promoted anisotropy within the cortical microtubule array (Figure 3Q; *p* = 0.005 for 3 μM GR24; *p* = 0.0375 for 25 μM GR24; *p* = 0.2381 for 3 μM TIS108).

Oppositely, light-grown mock-treated *max2-1* seedlings exhibited more biased arrays (Figures 3I,M) as compared to mock-treated Col-0 seedlings (Figures 3A,E), but treatments (Figures 3J–L,N–P) caused anisotropy reduction (Figure 3R; *p* = 0.0000 for 3 μM GR24, 25 μM GR24, and 3 μM TIS108). In this case, cortical microtubule anisotropy was 0.31 ± 0.1 after mock treatment (Figure 3R; mean ± SD; *N* = 35), but this was significantly reduced to 0.17 ± 0.07 after treatment with 3 μM GR24 (Figure 3R; mean ± SD; *N* = 31), to 0.14 ± 0.07 after 25 μM of GR25 (Figure 3R; mean ± SD; *N* = 61), and to 0.13 ± 0.06 for 3 μM TIS108 (Figure 3R; mean ± SD; *N* = 79).

**FIGURE 4:**
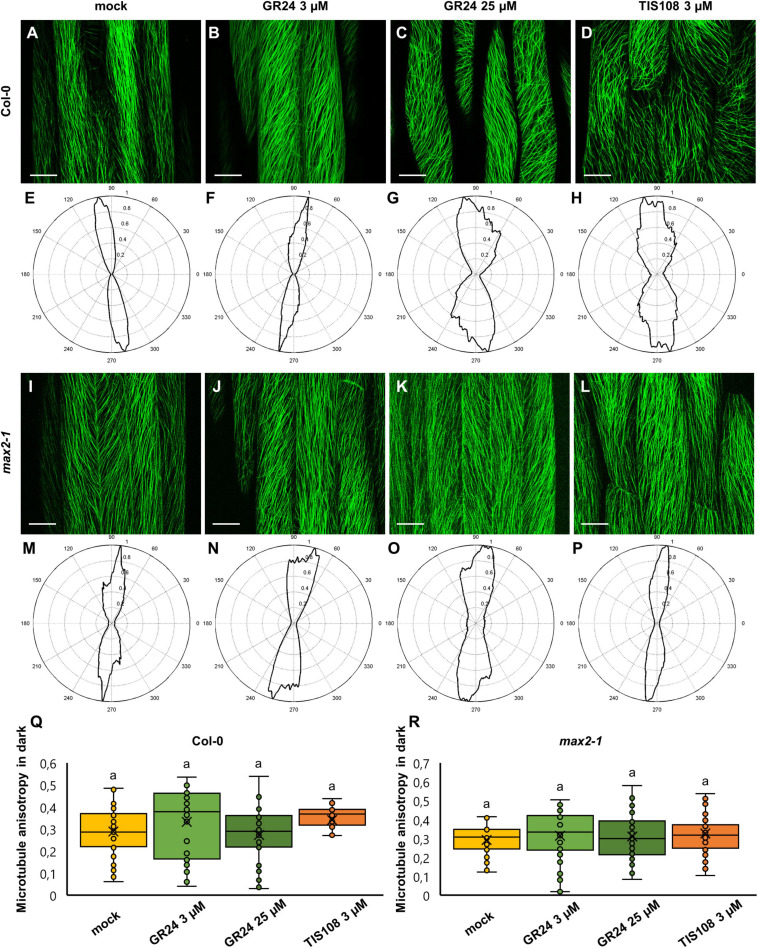
Assessment of microtubule organization in epidermal hypocotyl cells of etiolated seedlings of *Arabidopsis* Col-0 or the *max2-1* mutant expressing GFP-MBD construct in the presence or the synthetic strigolactone GR24 (3 μM and 25 μM) or the biosynthetic inhibitor of strigolactone production TIS108 (3 μM**). (A–D)** Overview of hypocotyl of Col-0 seedlings treated with mock **(A)**, 3 μM GR24 **(B)**, 25 μM GR24 **(C)**, 3 μM TIS108 **(D)**. **(E–H)** Cytospectre graphs of cortical microtubule distribution where panel **(E)** corresponds to panel **(A)**, panel **(F)** to panel **(B)**, panel **(G)** to panel **(C)**, and panel **(H)** to panel **(D)**. **(I–L)** Overview of hypocotyl of *max2-1* seedlings treated with mock **(I)**, 3 μM GR24 **(J)**, 25 μM GR24 **(K)**, 3 μM TIS108 **(L)**. **(M–P)** Cytospectre graphs of cortical microtubule distribution, where panel **(M)** corresponds to panel **(I)**, panel **(N)** to panel **(J)**, panel **(O)** to panel **(K)**, and panel **(P)** to panel **(L)**. **(Q)** Quantitative assessment of anisotropy of cortical microtubule organization in etiolated Col-0 after mock treatment and treatments with 3 μM GR24, 25 μM GR24, and 3 μM TIS108 (*N* ≥ 27; Welch’s ANOVA showed no statistically significant difference within the dataset; *F*(3, 143) = 3.1416, *p* = 0.030. **(R)** Quantitative assessment of anisotropy of cortical microtubule organization in etiolated *max2-1* after mock treatment and treatments with 3 μM GR24, 25 μM GR24, and 3 μM TIS108 (*N* ≥ 29; Welch’s ANOVA was followed with Scheffé’s test, but there was no statistically significant difference at the 0.01 probability level; results are in Supplementary Table 6). In all box plots, the average is presented by ×, median by the middle line, 1^st^ quartile by the bottom line, 3^rd^ quartile by the top line; the whiskers lie within the 1.5× interquartile range (defined from the 1^st^ to the 3^rd^ quartiles) while outliers are omitted. Scale bars: 20 μm.

In etiolated Col-0 seedlings, the orientation of cortical microtubules was more uniform as compared to light-grown ones. In all cases examined, microtubules were mostly parallel to each other and organized in longitudinal (e.g., Figure 4A), oblique (e.g., Figure 4B), and (rarely) transverse arrays (e.g., Figure 4C) with respect to the main cell axis. Notably, adjacent cells in the same sample may differ in the predominant microtubule orientation (e.g., Figures 4C,D). In mock-treated seedlings (Figures 4A,E), cortical microtubules were largely longitudinal, and this pattern seemed to be unaffected in seedlings treated with 3 μM GR24 (Figures 4B,F), but changed in case of 25 μM GR24 (Figures 4C,G) and 3 μM TIS108 (Figures 4D,H). Similarly, etiolated seedlings of *max2-1* mutant exhibited highly organized systems of parallel microtubules, at seemingly the same level of uniformity comparing to mock treatment (Figures 4I,M) or treatments with 3 μM GR24 (Figures 4J,N), 25 μM GR24 (Figures 4K,O), and 3 μM TIS108 (Figures 4L,P). Indeed, this observation was reflected to the level of cortical microtubule anisotropy, which in Col-0 was statistically similar between all cases (Figure 4Q; 0.29 ± 0.11, *N* = 27 for mock-treated Col-0; 0.33 ± 0.15, *N* = 42 for Col-0 treated with 3 μM GR24; 0.27 ± 0.12, *N* = 47 for Col-0 treated with 25 μM GR24; and 0.34 ± 0.09 for Col-0 treated with 3 μM TIS108; mean ± SD). In all cases, anisotropy of cortical microtubule organization was equally high in etiolated seedlings of the *max2-1* mutant, with minor variations within this group of treatments (Figure 4R). Thus, anisotropy values were 0.29 ± 0.08 for mock-treated seedlings (mean ± SD; *N* = 29), 0.31 ± 0.14 (mean ± SD; *N* = 54) after treatment with 3 μM GR24, 0.31 ± 0.12 (mean ± SD; *N* = 51) after 25 μM GR24, and 0.32 ± 0.11 (mean ± SD) after 3 μM TIS108 (Figure 4R).

In all cases, treatment of *max2-1* mutant seedlings promoted cortical microtubule disorganization at similar levels compared to mock-treated controls. With the exception of *max2-1* mutants, it seems that the anisotropic microtubule organization after GR24 and TIS108 treatments remains largely unaffected regardless of the illumination regime during seedling growth. Thus, in the case of *max2*-*1* mutants grown under light/dark, it appears that under control conditions (mock treatment) hypocotyl cells exhibit already highly organized arrays and all treatments uniformly promote cortical microtubule disorganization. Finally, it seems that etiolation promotes the biased organization of cortical microtubules irrespectively of treatments modulating strigolactone activity or synthesis.

### Alterations of Strigolactone Content Interfere With Microtubules Bundling

From the putative mechanisms underlying microtubules reorganization, bundling of adjacent microtubules, by microtubule-associated protein cross bridges, is one of the possibilities that can be related to the formation of uniform cortical arrays. In samples of fluorescently labeled microtubules, fluorescence is not distributed diffusely and evenly, and, in the image, there are areas with both pixels of high and low fluorescence intensities. The uniformity of fluorescence intensity distribution is reflected in a histogram depicting pixel frequency vs. fluorescence intensity (Supplementary Figure 3). When labeling is uniform, the histogram is close to a normal distribution with a central peak with the median value, symmetrically flanked by two tails (e.g., Supplementary Figure 3). When labeling is non-uniform, then the distribution of pixel frequencies per fluorescence intensity is skewed, and the peak is shifted left- or right-ward, which is linked to increasingly skewed distribution, depending on the degree of the non-uniformity of the signal. It must be noted that skewness of fluorescence intensity distribution is not related to the orientation of microtubules in the cortical cytoplasm (i.e., skewness is not an indicator of how cortical microtubules are organized). Although fluorescent labeling might appear to be uniform within an entire ROI by inspection, there are differences in the fluorescence intensity between different fluorescent structures, to which skewness is attributed. Such differences are not necessarily visible to the human eye, but they can be extrapolated from histogram analysis, which in this case were delivered automatically by the software used (Supplementary Figure 3). In this way, we quantified the skewness of fluorescence intensity distribution in hypocotyl cells of either Col-0 or *max2-1* untreated or treated with 3 μM and 25 μM GR24 as well as 3 μM TIS108 under light/dark regime or constant darkness.

**FIGURE 5:**
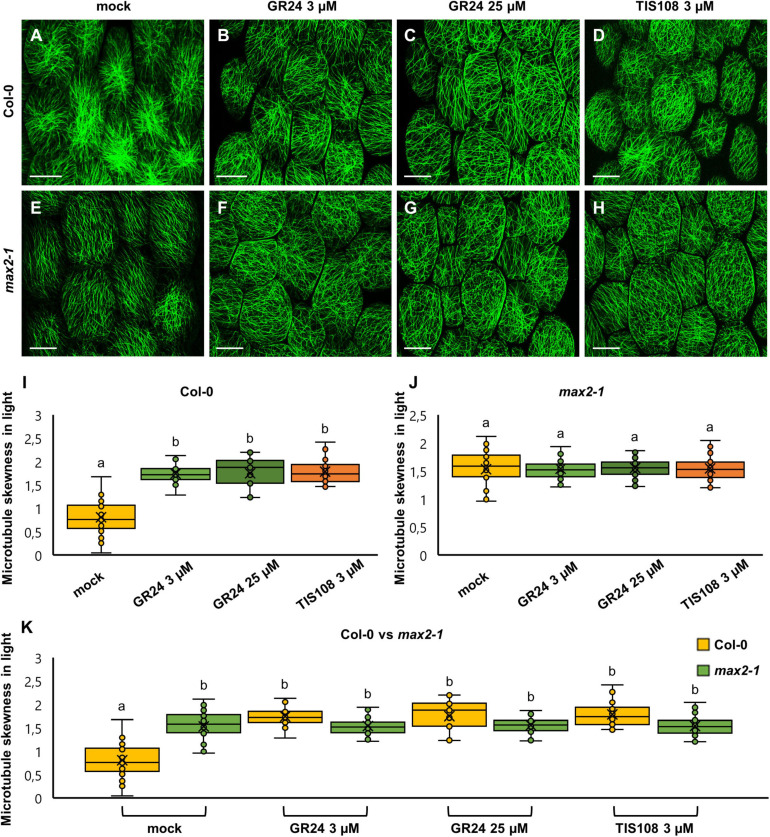
The skewness of fluorescence distribution of GFP-MBD-labeled microtubules of light-grown *Arabidopsis* Col-0 and *max2-1* epidermal hypocotyl cells in the presence or absence of the synthetic strigolactone GR24 (3 μM and 25 μM) or the biosynthetic inhibitor of strigolactone production TIS108 (3 μM). **(A–D)** Overviews of hypocotyl of Col-0 seedlings treated with mock **(A)**, 3 μM GR24 **(B)**, 25 μM GR24 **(C)**, 3 μM TIS108 **(D)**. **(E–H)** Overview of hypocotyl of *max2-1* seedlings treated with mock **(E)**, 3 μM GR24 **(F)**, 25 μM GR24 **(G)**, 3 μM TIS108 **(H)**. **(I,J)** Quantitative assessment of fluorescence distribution skewness, comparing Col-0 [**(I)**; *N* ≥ 17; Welch’s ANOVA was followed with Scheffé’s test; the statistical comparison is shown within groups sharing the same genotype; letters in the graph are shared by groups without statistically significant differences at the 0.01 probability level; results are in Supplementary Table 7] and *max2-1* [**(J)**; *N* ≥ 34; Welch’s ANOVA showed no statistically significant difference within the dataset; *F*(3, 161) = 0.0777, *p* = 0.9719]. **(K)** Collective quantification of fluorescence skewness comparing Col-0 and *max2-1* in a pairwise manner in all experimental conditions (*N* ≥ 17; two-way ANOVA was followed with Scheffé’s test; a statistical comparison is shown within groups sharing the same genotype; letters in the graph are shared by groups without statistically significant differences at the 0.001 probability level; results are in Supplementary Table 8). In all box plots, the average is presented by ×, median by the middle line, 1^st^ quartile by the bottom line, 3^rd^ quartile by the top line; the whiskers lie within the 1.5× interquartile range (defined from the 1^st^ to the 3^rd^ quartiles) while outliers are omitted. Scale bars: 20 μm.

In light-grown Col-0 cells, all treatments induced significantly higher skewness of the fluorescent signal as compared to mock-treated cells. In such mock-treated cells (Figure 5A), skewness was 0.80 ± 0.34 (Figure 5I; mean ± SD; *N* = 32), while after treatment with 3 μM GR24 (Figure 5B) it was 1.73 ± 0.23 (Figure 5I; mean ± SD; *N* = 31), and after treatments with 25 μM GR24 (Figure 5C) and 3 μM TIS108 (Figure 5D) it was 1.74 ± 0.34 (Figure 5I; mean ± SD; *N* = 17) and 1.78 ± 0.26 (Figure 5I; mean ± SD; *N* = 51), respectively. It is noteworthy that all treatments had a statistically significant effect as compared to the mock treatment (Figure 5I; *p* = 0.0000 for 3 μM GR24, 25 μM GR24, and 3 μM TIS108), though comparable to each other. The skewness of fluorescent signal from GFP-MBD line in *max2-1* mutant background was significantly higher compared to Col-0 (Figures 5E–H,J; *p* = 0.0000 for all treatments), but at comparable levels within all treatments in the *max2-1* group (Figure 5K). The increase of skewness in the Col-0 group (Figures 5A–D) might be relevant to the inducible increase of cortical microtubule anisotropy and may also underlie the intrinsically higher order of cortical microtubule organization of the *max2-1* mutant group (Figures 5 E–H). At the same time, it does not seem to correlate with the loosening of microtubule organization within the *max2-1* group (Figures 5E–H) after the interference with either strigolactone signaling or biosynthesis.

**FIGURE 6:**
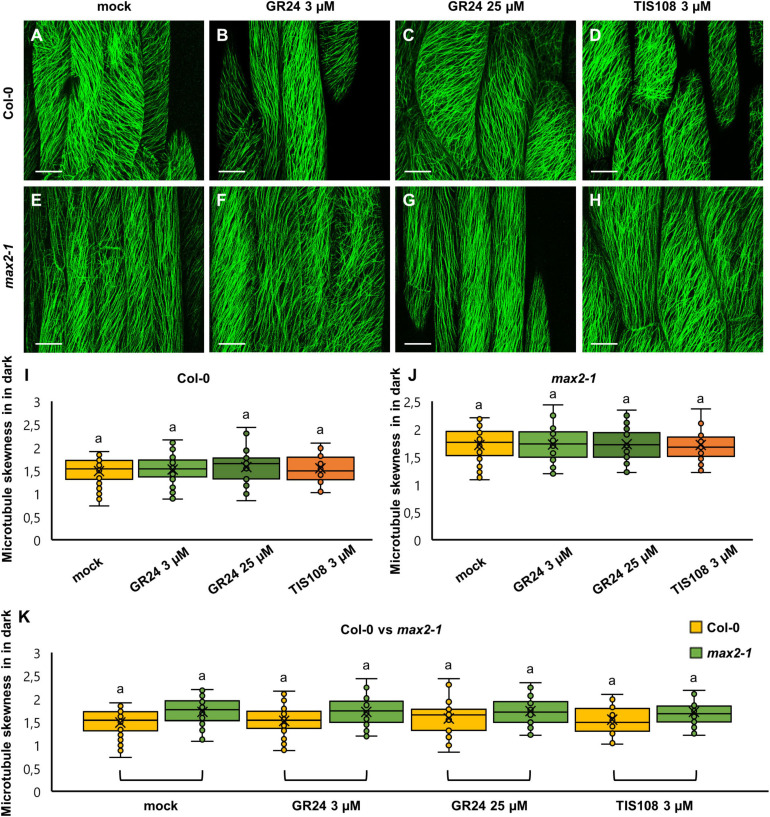
The skewness of fluorescence distribution of GFP-MBD-labeled microtubules of etiolated *Arabidopsis* Col-0 and *max2-1* epidermal hypocotyl cells in the presence or absence of the synthetic strigolactone GR24 (3 μM and 25 μM) or the biosynthetic inhibitor of strigolactone production TIS108 (3 μM). **(A–D)** Overviews of hypocotyl of Col-0 seedlings treated with mock **(A)**, 3 μM GR24 **(B)**, 25 μM GR24 **(C)**, 3 μM TIS108 **(D)**. **(E–H)** Overview of hypocotyl of *max2-1* seedlings treated with mock **(E)**, 3 μM GR24 **(F)**, 25 μM GR24 **(G)**, 3 μM TIS108 **(H)**. **(I,J)** Quantitative assessment of fluorescence distribution skewness comparing Col-0 [**(I)**; *N* ≥ 22; Welch’s ANOVA showed no statistically significant difference within the dataset; *F*(3, 161) = 0.4564, *p* = 0.7138] and *max2-1* [**(J)**; *N* ≥ 31; Welch’s ANOVA showed no statistically significant difference within the dataset; *F*(3, 151) = 0.0161, *p* = 0.9972]. **(K)** Collective quantification of fluorescence skewness comparing Col-0 and *max2-1* in a pairwise manner in all experimental conditions (*N* ≥ 22; two-way ANOVA was followed with Scheffé’s test, but there was no statistically significant difference at the 0.001 probability level; results are in Supplementary Table 9). In all box plots, the average is presented by ×, median by the middle line, 1^st^ quartile by the bottom line, 3^rd^ quartile by the top line; the whiskers lie within the 1.5× interquartile range (defined from the 1^st^ to the 3^rd^ quartiles) while outliers are omitted. Scale bars: 20 μm.

In dark-grown seedlings of either Col-0 or *max2-1* mutants, skewness of fluorescence distribution of GFP-MBD-labeled cortical microtubules was comparable between both groups with no statistically significant difference (Figure 6). In detail, mock-treated Col-0 cells showed a skewness value of 1.49 ± 0.3 (Figures 6A, I; mean ± SD; *N* = 57), while 1.52 ± 0.3 (Figures 6B,I; mean ± SD; *N* = 58)—after the treatment with 3 μM GR24, 1.58 ± 0.36 (Figures 6C,I; mean ± SD; *N* = 24)—after 25 μM GR24, and 1.55 ± 0.31 (Figures 6D,I; mean ± SD; *N* = 22)—after 3 μM TIS108. Similarly, etiolated mock-treated *max2-1* seedlings (Figure 6E) showed fluorescence skewness of 1.71 ± 0.31 (Figure 6J; mean ± SD; *N* = 31), 1.71 ± 0.30 (Figure 6J; mean ± SD; *N* = 39) after treatment with 3 μM GR24 (Figure 6F), 1.73 ± 0.29 (Figure 6J; mean ± SD; *N* = 34) after 25 μM GR24 (Figure 6G), and 1.71 ± 0.35 (Figure 6J; mean ± SD; *N* = 47) after 3 μM TIS108 (Figure 6H). Although skewness values of *max2-1* etiolated seedlings were consistently higher than those of the Col-0 group, the differences inferred were not significant (Figure 6K). These results suggest that chemical interference with strigolactone signaling or synthesis affects microtubule bundling in light rather than in dark.

### The Dynamics of Cortical Microtubules Are Modulated by GR24

Microtubule dynamics were followed by means of time-lapsed SIM in hypocotyl cells of dark-grown Col-0 or *max2-1* mutants, both stably expressing the GFP-MBD microtubule marker. Using a frame rate of *ca.* 0.4 frames per second (fps) it was possible to record time series of end-wise length excursions of individual or bundled microtubules and quantify measures of plus-end dynamic instability using appropriately generated kymographs.

**FIGURE 7:**
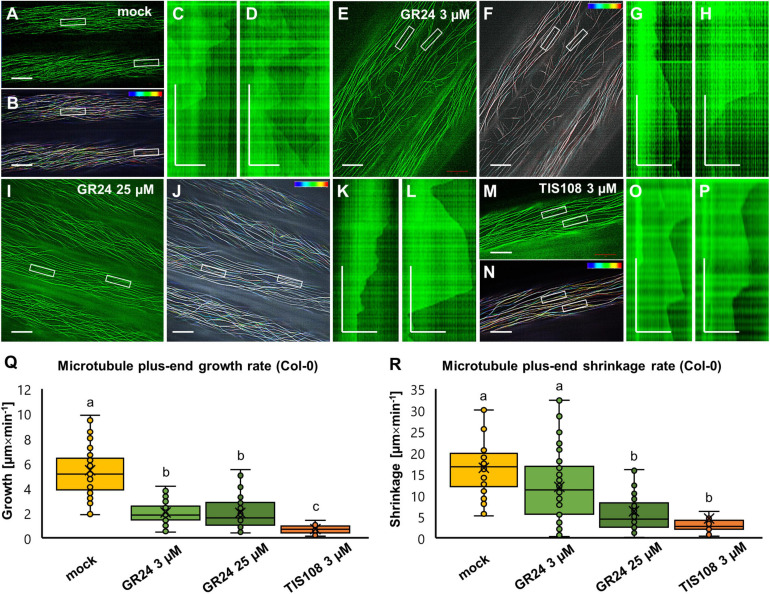
Analysis of microtubule dynamics of *Arabidopsis* Col-0 expressing the GFP-MBD microtubule marker in the presence or absence of the synthetic strigolactone GR24 (3 μM and 25 μM) or the biosynthetic inhibitor of strigolactone production TIS108 (3 μM). **(A,B)** Overview **(A)** and color-coded projection **(B)** of the time series corresponding to mock-treated Col-0 (see Supplementary Movie 1). **(C,D)** Two kymographs showing length fluctuations of the left **(C)** and the right **(D)** boxed areas of panel **(A,B)**. **(E,F)** Overview **(E)** and color-coded projection **(F)** of the time series corresponding to Col-0 treated with 3 μM GR24 (Supplementary Movie 2). **(G,H)** Two representative kymographs from boxed areas 1 and 2 of panels **(E,F)** showing decelerated and sustainable growth and shrinkage. **(I,J)** Overview **(I)** and color-coded projection **(J)** of the time series corresponding to Col-0 treated with 25 μM GR24 (Supplementary Movie 3). **(K,L)** Two representative kymographs from boxed areas 1 and 2 of panels **(I,J)** showing prolonged growth and shrinkage at lower rates compared to mock-treated cells. **(M,N)** Overview **(M)** and color-coded projection **(N)** of the time series corresponding to Col-0 treated with 3 μM TIS108 (Supplementary Movie 4). **(O,P)** Two representative kymographs from boxed areas 1 and 2 of panels **(M,N)** showing prolonged growth and shrinkage at lower rates compared to mock-treated cells. **(Q,R)** Quantitative assessment of microtubule growth [**(Q)**; *N* ≥ 33; Welch’s ANOVA was followed with Scheffé’s test; a statistical comparison is shown within groups sharing the same genotype; letters in the graph are shared by groups without statistically significant differences at the 0.01 probability level; results are in Supplementary Table 10] and shrinkage [**(R)**; *N* ≥ 28; Welch’s ANOVA was followed with Scheffé’s test; a statistical comparison is shown within groups sharing the same genotype; letters in the graph are shared by groups without statistically significant differences at the 0.01 probability level; results are in Supplementary Table 11] of Col-0 GFP-MBD-labeled microtubule in all experimental conditions. In all box plots, the average is presented by ×, median by the middle line, 1^st^ quartile by the bottom line, 3^rd^ quartile by the top line; the whiskers lie within the 1.5× interquartile range (defined from the 1^st^ to the 3^rd^ quartiles) while outliers are omitted. Scale bars: 10 μm **(A,B,E,F,I,J,M,N)**; 5 μm **(C,D,G,H,K,L,O,P)**. All time bars correspond to 2 min.

In mock-treated Col-0 hypocotyl epidermal cells expressing GFP-MBD (Figures 7A,B and Supplementary Movie 1), plus end growth and shrinkage rates as well as catastrophe and rescue frequencies measured from appropriate kymographs (Figures 7C,D) were within previously published values (Komis et al., 2014). Briefly, the average growth rate was 5.46 ± 2.76 μm × min^–1^ (mean ± SD; *N* = 53 microtubule ends), while the average shrinkage rate was 16.48 ± 6.25 μm × min^–1^ (mean ± SD; *N* = 50 microtubule ends). Furthermore, catastrophe frequency was 0.0122 events × s^–1^, while rescue frequency was 0.0512 events × s^–1^. In both cases of GR24 treatment (3 μM and 25 μM) plus-end microtubule dynamics were considerably slowed during both growth and shrinkage. At the concentration of 3 μM (Figures 7E–H and Supplementary Movie 2) the average growth rate was 2.05 ± 0.96 μm × min^–1^ (mean ± SD; *N* = 50 microtubule ends), and the average shrinkage rate was 12 ± 8 μm × min^–1^ (mean ± SD; *N* = 50 microtubule ends). The catastrophe frequency was 0.0082 events × s^–1^, while rescue frequency was 0.0332 events × s^–1^. At 25 μM (Figures 7I–L and Supplementary Movie 3), the average growth rate was 2.01 ± 1.23 μm × min^–1^ (mean ± SD; *N* = 59 microtubule ends), and the average shrinkage rate was 6.23 ± 5.46 μm × min^–1^ (mean ± SD; *N* = 42 microtubule ends). The catastrophe frequency was 0.0078 events × s^–1^, while rescue frequency was 0.0288 events × s^–1^. The biosynthetic inhibitor TIS108 (Figures 7M–P and Supplementary Movie 4) strongly inhibited microtubule plus end dynamic parameters. In general, the average growth rate was 0.70 ± 0.32 μm × min^–1^ (mean ± SD; *N* = 33 microtubule ends) and the average shrinkage rate was 4.59 ± 5.42 μm × min^–1^ (mean ± SD; *N* = 28 microtubule ends). The catastrophe frequency was 0.0077 events × s^–1^ while the rescue frequency was 0.0255 events × s^–1^.

By comparison to mock-treated cells, both parameters of microtubule dynamics were in most cases significantly reduced in all treatments tested (Figure 7Q for growth rate and Figure 7R for shrinkage rate). In terms of growth rate (Figure 7Q) both concentrations of GR24 showed comparable reduction as compared to mock treatment, while growth rates were even more reduced in the case of treatment with TIS108 (Figure 7Q; *p* = 0.0000 for 3 μM GR24; 25 μM GR24, and 3 μM TIS108). Shrinkage rates were also reduced in all treatments (Figure 7R; *p* = 0.0108 for 3 μM GR24; and *p* = 0.0000 for both 25 μM GR24 and 3 μM TIS108).

**FIGURE 8:**
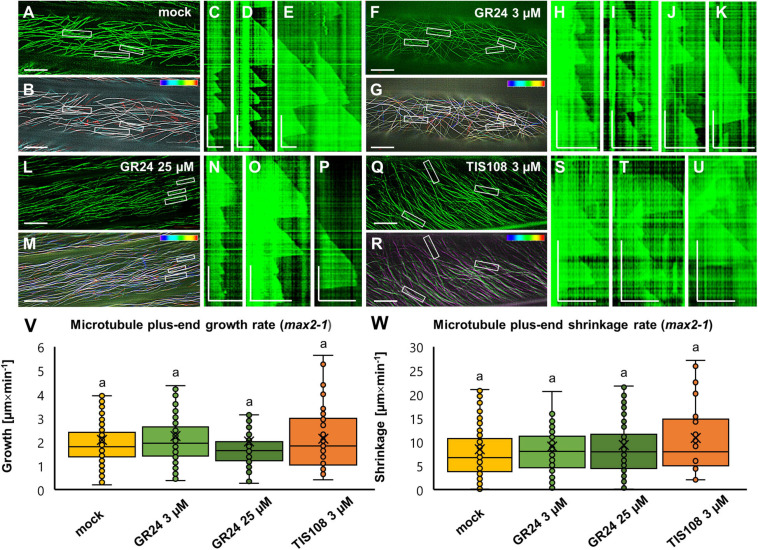
Analysis of microtubule dynamics of *Arabidopsis max2-1* mutant expressing the GFP-MBD microtubule marker in the presence or absence of the synthetic strigolactone GR24 (3 μM and 25 μM) or the biosynthetic inhibitor of strigolactone production TIS108 (3 μM). **(A,B)** Overview (A) and color-coded projection **(B)** of the time series corresponding to mock-treated *max2-1* (Supplementary Movie 5). **(C,D,E)** Three kymographs showing microtubule length fluctuations corresponding to boxed areas 1,2,3 of panels **(A,B)**, indicative of slower and prolonged growth and shrinkage compared to Col-0. **(F,G)** Overview **(F)** and color-coded projection **(G)** of the time series corresponding to *max2-1* treated with 3 μM GR24 (Supplementary Movie 6). **(H–K)** Four representative kymographs from boxed areas 1,2,3 and 4 of panels **(F,G)** showing similar microtubule dynamics as in mock-treated cells. **(L,M)** Overview **(L)** and color-coded projection **(M)** of the time series corresponding to *max2-1* treated with 25 μM GR24 (Supplementary Movie 7). **(N–P)** Three representative kymographs from boxed areas 1, 2, and 3 of panels **(L,M)** showing comparable growth and shrinkage to mock-treated cells. **(Q,R)** Overview **(Q)** and color-coded projection **(R)** of the time series corresponding to *max2-1* treated with 3 μM TIS108 (Supplementary Movie 8). **(S–U)** Three representative kymographs from boxed areas 1, 2, and 3 of panels **(Q,R)**. **(V,W)** Quantitative assessment of microtubule growth [**(V)**; *N* ≥ 41; Welch’s ANOVA showed no statistically significant difference within the dataset; *F*(3, 601) = 0.6081, *p* = 0.6106] and shrinkage [**(W)**; *N* ≥ 20; Welch’s ANOVA showed no statistically significant difference within the dataset; *F*(3, 333) = 80.2659, *p* = 0.6649] of GFP-MBD labeled microtubule in all experimental conditions. In all box plots, the average is presented by ×, median by the middle line, 1^st^ quartile by the bottom line, 3^rd^ quartile by the top line; the whiskers lie within the 1.5× interquartile range (defined from the 1^st^ to the 3^rd^ quartiles) while outliers are omitted. Scale bars: 10 μm **(A,B,F,G,L,M,Q,R)**; 5 μm **(H–K,N–P,S–U)**; 2 μm **(C–E)**. All time bars correspond to 2 min.

The most striking feature of GFP-MBD-labeled microtubules in the *max2-1* mutant was the significantly lower growth rate and most importantly the long-sustained growth periods of nearly every microtubule examined. The prolonged elongation of cortical microtubules was clearly evident in mock-treated *max2-1* seedlings (Figures 8A–E and Supplementary Movie 5) with the average growth rate being 2.09 ± 1.27 μm × min^–1^ (mean ± SD; *N* = 32 microtubule ends) and the average shrinkage rate being 8.48 ± 7.06 μm × min^–1^ (mean ± SD; *N* = 54 microtubule ends). In such seedlings, the catastrophe frequency was 0.0087 events × s^–1^ and the rescue frequency—0.0266 events × s^–1^. However, the exogenous application of GR24 at either 3 or 25 μM, or the treatment with TIS108, had no effect on any parameter of microtubule dynamics compared to mock-treated *max2-1* cells. Briefly, in *max2-1* seedlings treated with 3 μM GR24 (Figures 8F–K and Supplementary Movie 6), the average growth rate was 2.25 ± 1.35 μm × min^–1^ (mean ± SD; *N* = 134 microtubule ends) and the average shrinkage rate was 9.03 ± 7.38 μm × min^–1^ (mean ± SD; *N* = 87 microtubule ends). In turn, catastrophe frequency was 0.0071 events × s^–1^ while rescue frequency was 0.0301 events × s^–1^. A similar case was the situation of *max2-1* seedlings treated with 25 μM (Figures 8L–P and Supplementary Movie 7), where average growth was measured at 2.02 ± 1.73 μm × min^–1^ (mean ± SD; *N* = 167 microtubule ends), and average shrinkage rate was calculated to be 9.34 ± 6.76 μm × min^–1^ (mean ± SD; *N* = 92 microtubule ends). The catastrophe and rescue frequencies were 0.0081 events × s^–1^ and 0.0264 events × s^–1^, respectively. As in the case of GR24, *max2-1* mutants were relatively insensitive to TIS108 treatment as well (Figures 8Q–U and Supplementary Movie 8). Therefore, the growth rate was 2.13 ± 1.24 μm × min^–1^ (mean ± SD; *N* = 41 microtubule ends) and the shrinkage rate was 10.84 ± 7.70 μm × min^–1^ (mean ± SD; *N* = 20 microtubule ends). Catastrophe and rescue frequencies were 0.0083 events × s^–1^ and 0.0222 events × s^–1^, respectively. As mentioned before, treatments had no significant effect on neither growth (Figure 8V) nor shrinkage (Figure 8W) within the *max2-1* group.

Uniformly, growth rates in Col-0 group were reduced compared to mock treatment in a similar manner to the growth rates of *max2-1* (Supplementary Figure 4A; *p* = 0.0000 for 3 μM GR24, 25 μM GR24, and 3 μM TIS108). Reductions in shrinkage rates showed higher variability either comparing different experimental conditions within the Col-0 group or by comparing the Col-0 group with the *max2-1* group (Supplementary Figure 4B; *p* = 0.1732 for 3 μM GR24; and *p* = 0.0000 for both 25 μM GR24 and 3 μM TIS108).

Conclusively, the aforementioned results suggest that alterations in strigolactone signaling either by chemical (GR24 and TIS108 treatments) or genetic (*max2-1* mutant) interference, uniformly reduce microtubule dynamicity and likely promote microtubule longevity, as evidenced by the considerably lower catastrophe frequencies observed.

## Discussion

**FIGURE 9:**
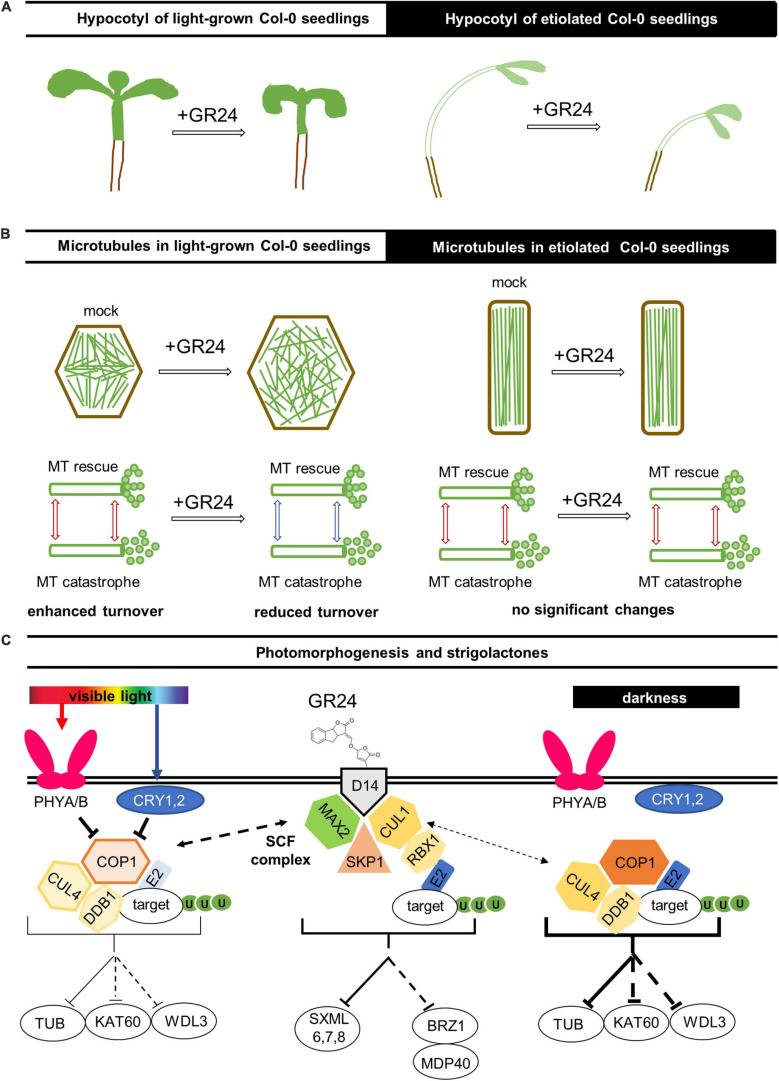
Hypothetical model of light-dependent strigolactone effects on microtubules in *Arabidopsis*. **(A)** Regarding the overall hypocotyl phenotype, the strigolactone treatment inhibits hypocotyl growth and causes radial expansion of epidermal cells in light-grown seedlings; however, the dark-grown ones were more resistant to the changes of strigolactone content. **(B)** In light-grown seedlings the treatment with GR24 leads to changes in microtubule organization and dynamics: (1) more pronounced randomization of cortical microtubule array; (2) increased microtubule bundling and stabilization; (3) reduced microtubule dynamicity and likely promoted microtubule longevity. On the other hand, no significant microtubule changes were noted after similar treatments in etiolated seedlings as the trend is to maintain highly organized systems of parallel microtubules. **(C)** Red and blue lights are perceived by PHYTOCHROMES A and B (PHYA/B) and CRYPTOCHROMES 1 and 2 (CRY1/2), respectively, which inhibit the E3 ligase complex consisting of CONSTITUTIVE PHOTOMORPHOGENIC 1 (COP1), CULLIN4 (CUL4), and DAMAGE-BINDING PROTEIN 1 (DDB1). This E3 ligase complex directs the transfer of ubiquitin (U) from an E2 ligase onto targets, which generally leads to their degradation by proteasomes. Bellow the complex, known (solid line) and putative (dotted line) targets are shown, specifically TUBULIN (TUB), KATANIN 60 (KAT60), and WAVE-DAMPENED 2-LIKE 3 (WDL3). The function of this E3 ligase complex is more prominent under darkness when it is not inhibited by PHYA/B and CRY1/2. It has been previously proposed that COP1 might be regulated by the SKP1-CULLIN-F-BOX (SCF) complex containing an F-box protein MORE AUXILARY GROWTH 2 (MAX2). This SCF complex consists of MAX2, hydrophobic scaffold protein CULLIN1 (CUL1), S-phase kinase-associated protein 1 (SKP1), and E3 ubiquitin-protein ligase RING-BOX1 (RBX1); it also functions as an E3 ligase; known (solid line) and putative (dotted line) targets are shown, namely, SUPPRESSOR OF MORE AXILLARY GROWTH2-LIKE (SMXL) proteins and transcriptional repressor BRASSINAZOLE-RESISTANT 1 (BZR1), which is involved in the regulation of microtubules *via* the MICROTUBULE DESTABILIZING PROTEIN40 (MDP40). SCF complex is activated by artificial strigolactones GR24+ binding to an α/β-hydrolase D14, strigolactone-specific receptor.

In the present study, we followed the long-term effects of exogenously applied strigolactone synthetic analog GR24 and a strigolactone biosynthesis inhibitor TIS108 on the growth of Arabidopsis hypocotyls. Given the relationship between strigolactone effects and light conditions (Xie et al., 2020), our study was extended to seedlings exposed to periodic illumination, or exclusively grown in the dark. We studied wild-type Col-0 Arabidopsis seedlings and, furthermore, applied the same set of treatments to *max2-1*, an Arabidopsis mutant corresponding to the F-box protein MAX2, which is an integral part of the perception mechanism of both strigolactones and karrikins (Nelson et al., 2011; Waters and Smith, 2013; De Cuyper et al., 2017; Villaécija-Aguilar et al., 2019; Swarbreck et al., 2020; Wang et al., 2020b). Both Col-0 and *max2-1* seedlings were transformed with fluorescent microtubule markers (GFP-MBD and GFP-TUA6), aiming to examine whether the effects of GR24 and TIS108 on hypocotyl growth might have been related to alterations in cortical microtubule organization and/or dynamics. In summary, GR24 and TIS108 induced changes in the global properties of cortical microtubule arrays reflected in their degree of anisotropy and bundling and such changes were more evident in light/dark-grown seedlings than in etiolated ones. Microtubule growth and shrinkage rates were robustly reduced in etiolated Col-0 after all treatments examined while the inherently lower dynamic parameters of *max2-1* seedlings remained unaffected. Hence, the shaping of hypocotyl architecture is defined by both strigolactones and light, and microtubule cytoskeleton rearrangement might be important for this multidimensional process (Figures 9A,B).

Being produced mainly in the roots (Foo et al., 2013), strigolactones adjust both shoot (Gomez-Roldan et al., 2008; Umehara et al., 2008) and root (Ruyter-Spira et al., 2011) development in vascular plants as well as in moss caulonema (Hoffmann et al., 2014) in response to changing environmental conditions. Early grafting experiments showed that strigolactones are transported from roots to shoot in the xylem of Arabidopsis and tomato, which provided insight into strigolactone signaling regulation *via* their localization and transport (Kohlen et al., 2011). Strigolactones may either enhance or inhibit organ size and number depending on the organ (Delaux et al., 2012; Hoffmann et al., 2014). In this study, the role of strigolactones in light-dependent shaping of hypocotyl architecture *via* microtubule cytoskeleton rearrangement was addressed, and putative signaling pathways are summarized in Figure 9C.

The exogenous application of a synthetic strigolactone, GR24 (Umehara et al., 2008) and an inhibitor of endogenous strigolactone production, TIS108, a potent triazole-containing inhibitor of cytochrome P450 monooxygenases (Ito et al., 2010, 2011, 2013), resulted in hypocotyl growth alterations in both Col-0 and a strigolactone perception mutant in *MAX2*, a gene encoding a member of the F-box leucine-rich repeat protein family, which is likely the substrate recognition subunit of SCF ubiquitin E3 ligase for targeted proteolysis at the proteasome (Stirnberg et al., 2002; Wang et al., 2013, 2015). Alleles of *max2* mutant are rendered insensitive to exogenous strigolactone application in phenomena such as strigolactone-induced inhibition of hypocotyl elongation (Jia et al., 2014; Wang et al., 2020b), suppression of shoot branching (Wang et al., 2013; Liu et al., 2014; Li et al., 2016) and lateral root formation (Ruyter-Spira et al., 2011; Li et al., 2016). Moreover, the function of MAX2 is associated with photomorphogenesis in angiosperms (Shen et al., 2007; Nelson et al., 2011; Waters and Smith, 2013) and mosses (Lopez-Obando et al., 2018). MAX2 is also involved in KARRIKIN-INSENSITIVE2 (KAI2)-ligand pathway (Nelson et al., 2011). Moreover, the racemic mixtures of two stereoisomers in *rac*-GR24 overlap with the KAI2-mediated karrikin signaling pathway (De Cuyper et al., 2017).

Previous studies on the effects of exogenous strigolactones on vegetative growth have shown that compounds such as GR24 exert an inhibitory role on the skotomorphogenic elongation of the hypocotyl and on branching processes of either the shoot or the root culminating in the reduction of tillering and lateral root formation among others (Ruyter-Spira et al., 2013; Jiang et al., 2016; Sun et al., 2019). Additionally, the input of Arabidopsis genotype needs to be taken into account, since strigolactone mutants on Col-0 and Ler background differ in terms of hypocotyl elongation (Nelson et al., 2011; Waters and Smith, 2013).

The effects of strigolactone signaling manipulation were conspicuously evident in light-grown and, to a lesser extent, in etiolated seedlings. Indeed, previous studies have shown that exogenous strigolactones application halts hypocotyl elongation of light-grown seedlings in a dose-dependent manner, being notable at even lower concentrations than the ones used herein (e.g., at 100 nM; Jia et al., 2014). Importantly, *max2* mutant alleles showed negligible response at low concentrations of exogenous strigolactones and exhibited inhibition of hypocotyl elongation at concentrations exceeding 25 μM (Jia et al., 2014). These results corroborate the previous studies on the synergy between strigolactones and light perception (Brewer et al., 2013), involving a correlation of strigolactone sensing with both phytochrome and cryptochrome light-dependent signaling (Jia et al., 2014).

Diffuse organ growth (i.e., elongation or lateral expansion) is conditionally regulated by physical or hormonal signals and involves the positional control of cellulose microfibril deposition. In this sense, cortical microtubules have been repeatedly shown to underlie cell and organ growth rate and directionality as shown in the case of light (e.g., Sambade et al., 2012; Lindeboom et al., 2013; Ma et al., 2018), mechanical stimulation (Louveaux et al., 2016; Takatani et al., 2020), and hormonal cues including ethylene (Ma et al., 2018; Wang et al., 2020c), brassinosteroids (Wang et al., 2012), auxins (True and Shaw, 2020) and gibberellins (Locascio et al., 2013; Vineyard et al., 2013).

In light of the above, the present study was extended to address whether manipulation of strigolactone signaling could be related to cytoskeletal remodeling. Thus, the organization and the dynamics of cortical microtubules were studied in appropriate fluorescent marker lines of both Col-0 and *max2-1* mutants. In terms of organization, exogenous strigolactones application and inhibition of endogenous strigolactone biosynthesis under standard light/dark exposure just slightly reoriented cortical microtubules relatively to the cell longitudinal axis in Col-0, but had a prominent effect in *max2-1* mutants, promoting randomization of the cortical array. The higher intrinsic anisotropy of cortical microtubules of mock-treated hypocotyl epidermal cells of *max2-1* mutant seedlings might explain the effects of GR24 and TIS108. Microtubule bundling was enhanced after all treatments in Col-0 but remained unchanged in *max2-1* mutants, which seemingly exhibited a higher level of bundling than Col-0 in all circumstances. Notably, such microtubule organization features as ordering and bundling remained fairly unresponsive to the chemical treatments in etiolated seedlings of both Col-0 and *max2-1*. Microtubule dynamics were considerably lowered after chemical manipulation of strigolactone signaling in Col-0, while the inherently lower microtubule dynamics of *max2-1* remained unresponsive to GR24 and TIS108.

Owing to the previous connection of strigolactones with phytochrome and cryptochrome light perception pathways, the differential responses of cortical microtubule to strigolactone content alterations under light or dark growth conditions is expected. Earlier studies have already demonstrated the interdependence between phytochromes and light-induced microtubule reorientation (Fischer and Schopfer, 1997), while more recently, the reorientation of cortical microtubule under blue light stimulation was attributed to activation of KATANIN-mediated microtubule severing *via* the activation of the PHOT1 and PHOT2 phototropin photoreceptors (Lindeboom et al., 2013).

At present, the molecular components responsible for strigolactone-mediated suppression of microtubule dynamics in Arabidopsis remain unknown. Its putative mechanisms are summarized in the speculative hypothetical model of the interplay of light- and strigolactone-induced pathways, which regulates the organization and dynamics of cortical microtubule resulting in the subsequent changes of hypocotyl growth and morphology (Figure 9C). The initial perception of strigolactones in the karrikin-independent pathway is provided by α/β hydrolase AtD14 (Seto et al., 2019), being activated by its binding with the ligand and able to form complex with MAX2 (reviewed by Kumar et al., 2015b; Wang et al., 2020b). Upon the assembly of the SCF complex, including CULLIN1 (CUL1), Skp1 (S-phase kinase-associated protein 1), and E3 ubiquitin-protein ligase RING-BOX1 (RBX1), it directs ubiquitin transfer from an E2 ligase onto target proteins, which leads to their proteasome degradation. SCF complex containing MAX2 is known to affect plant development *via* the degradation of SUPPRESSOR OF MORE AXILLARY GROWTH2-LIKE (SMXL) proteins (Wang et al., 2020b). Another putative target protein for MAX2-mediated ubiquitination is one of the key transcription factors of the brassinosteroid pathway, namely BRASSINAZOLE-RESISTANT 1 (BZR1), which directly targets and upregulates MICROTUBULE DESTABILIZING PROTEIN40 (MDP40), a positive regulator of hypocotyl cell elongation by altering the stability of cortical microtubules (Wang et al., 2012). The more pronounced randomization of cortical microtubule array, increased microtubule bundling and stabilization as well as reduced microtubule dynamicity and likely promoted microtubule longevity leading to the stalled hypocotyl elongation and radial swelling of epidermal cells might be regulated by the BZR1-MDP40 pathway branch as well.

Alternatively, the strigolactone pathway might interplay with the light-induced one *via* the different types of an E3 ligase complex consisting of CUL4, DNA DAMAGE-BINDING PROTEIN 1 (DDB1), and CONSTITUTIVE PHOTOMORPHOGENIC 1 (COP1). The COP1 is subjected to regulation by PHYTOCHROME A and B (PHYA/B), photoreceptors of red light, and CRYPTOCHROMES 1 and 2 (CRY1/2), photoreceptors of blue light (Podolec and Ulm, 2018). It has been previously proposed that COP1 might be regulated by the SCF complex containing MAX2 (Jia et al., 2014). Moreover, a E3 ligase complex, including COP1, might target tubulin (Khanna et al., 2014) as well as the proteins involved in cytoskeleton regulation such as phototropin-stimulated microtubule-severing protein katanin (Lindeboom et al., 2013) and microtubule-associated protein WAVE-DAMPENED 2-LIKE 3 (WDL3) that binds to, bundles and stabilizes microtubules (Liu et al., 2013; Lian et al., 2017; Figure 9C).

However, another plausible explanation may refer to the physiological differences between hypocotyls and roots, especially in relation to the interplay between strigolactone signaling and light perception. As mentioned previously, light-induced microtubule reorientations in aboveground tissues have been shown to correlate with phytochrome (Zandomeni and Schopfer, 1993; Fischer and Schopfer, 1997) and phototropin (Lindeboom et al., 2013) signaling. The roots are also not indifferent to light, since dim light gradients may form at shallow depths of the soil and probably express specialized photoreceptors responsive to low illumination rates especially at the blue wavelength range (Galen et al., 2007; Wan et al., 2019). Differences in photoreception between aboveground and soil-residing plant parts may explain discrepancies in the cellular responses to exogenous strigolactones or strigolactone biosynthesis inhibitors and this is a matter that deserves to be followed up.

Although TIS108 is an inhibitor of P450 cytochrome monooxygenases and thus supposed to be an antagonist of strigolactone function, previous reports have confirmed its inhibitory effect to hypocotyl elongation (Kawada et al., 2019, 2020). On this basis, the follow-up effects of TIS108 on cortical microtubule organization and dynamics are in line with its observed effects on hypocotyl growth. Since the effects of TIS108 are also differentiated between light-grown and etiolated seedlings, it is likely that the TIS108-induced cytoskeletal remodeling is also associated to imbalances in strigolactone signaling. However, TIS108 might be not completely specific to strigolactone biosynthesis, since they target other strigolactone-unrelated CYP450 (Kawada et al., 2020). Hence, future studies have to be supplemented by the use of triflumizole, a novel lead compound for strigolactone biosynthesis (Kawada et al., 2020) as well as *Atmax3* and/or *Atmax4* strigolactone synthesis mutants, which could both be chemically complemented by the addition of external strigolactones (Booker et al., 2004).

It can be assumed that karrikins might also affect the plant cytoskeleton. Therefore, the use of commercially available exogenous karrikins, KAR1 and KAR2, which affect hypocotyl elongation (Nelson et al., 2011), as well as *kai2 (karrikin-insensitive2)* mutant (Villaécija-Aguilar et al., 2019), will be beneficial for experimental testing this hypothesis.

In conclusion, the present study highlights the significance of cytoskeleton remodeling in the process of GR24-mediated inhibition of hypocotyl growth, and reveals the differential regulation of both microtubule organization and dynamics by synthetic strigolactone GR24 at different illumination regimes.

## Data Availability Statement

The original contributions presented in the study are included in the article/Supplementary Material, further inquiries can be directed to the corresponding author/s.

## Author Contributions

YK and JŠ designed experiments alongside contributions from GK. YK and SH carried out all image acquisitions with the help of GK and MO. YK, SH, and GK carried out all post-acquisition image processing. YK and SH carried out all hypocotyl length and width measurements. GK acquired all necessary measurements and analyzed all data related to microtubule organization and dynamics. TV carried out statistical analyses. TP synthesized GR24. YK and GK drafted the manuscript. GK compiled all figures with input from YK, TV, and JŠ. JŠ provided funding and infrastructure. All authors contributed to the article and approved the submitted version.

## Conflict of Interest

The authors declare that the research was conducted in the absence of any commercial or financial relationships that could be construed as a potential conflict of interest.

## Footnotes

1 https://imagescience.org/meijering/software/neuronj/

2 imagej.nih.gov/ij/

## Supplementary Material

Supplementary Figure 1 Measurements of light-exposed **(A)** and etiolated **(B)** Col-0 and *max2-1* hypocotyl length after treatment with GR24 or TIS108 (two-way ANOVA with Scheffé’s *post hoc* test; common letters denote groups without statistically significant differences at the 0.001 probability level; Supplementary Tables 1, 3). These measurements of hypocotyl growth were performed using a NeuronJ plugin for ImageJ (Meijering, 2010), as an alternative and complementary measuring approach.

Supplementary Figure 2 Quantitative extent of the effect of GR24 interference in hypocotyl elongation of light-grown Col-0 **(A)**, light-grown *max2-1* **(B)**, and etiolated Col-0 **(C)** and *max2-1* **(D)** seedlings. **p* < 0.05; ****p* < 0.001 according to Student’s *t*-test.

Supplementary Figure 3 Example of skewness determination as an indicator of microtubule bundling, based on Figure 3B. **(A)** Overview (same as Figure 3B). **(B)** Magnified view of the ROI corresponding to the bottom right boxed area of panel **(A)**. **(C)** Magnified view of the ROI corresponding to the top left boxed area of panel **(A)**. **(D–F)** Histograms of fluorescence intensity frequencies corresponding to panel **(A)** in panel **(D)**, to panel **(B)** in panel **(E)** and to panel **(C)** in panel **(F)**. Scale bars: 20 μm.

Supplementary Figure 4 Pairwise comparison of Col-0 and *max2-1* microtubule plus-end growth **(A)** (*N* ≥ 33; two-way ANOVA was followed with Scheffé’s test; statistical comparison is shown within groups sharing the same genotype; letters in the graph are shared by groups without statistically significant differences at the 0.001 probability level; results are in Supplementary Table 12) and shrinkage **(B)** (*N* ≥ 20; two-way ANOVA was followed with Scheffé’s test, a statistical comparison is shown within groups sharing the same genotype; letters in the graph are shared by groups without statistically significant differences at the 0.001 probability level; results are in Supplementary Table 13) under all experimental conditions used herein. In all box plots, the average is presented by ×, median by the middle line, 1^st^ quartile by the bottom line, 3^rd^ quartile by the top line; the whiskers lie within the 1.5× interquartile range (defined from the 1^st^ to the 3^rd^ quartiles) while outliers are omitted.

Supplementary Figure 5 Assessment of microtubule organization in epidermal hypocotyl cells of light-grown seedlings of Arabidopsis Col-0 wild type or the *max2-1* mutant expressing GFP-TUA6 construct in the presence or absence of GR24 synthetic strigolactone (3 μM and 25 μM) or the biosynthetic inhibitor of strigolactone production TIS108 (3 μM). **(A–D)** Overview of hypocotyl of Col-0 seedlings treated with solvent alone [mock; **(A)**], 3 μM of GR24 **(B)**, 25 μM of GR24 **(C)**, and 3 μM of TIS108 **(D)**. **(E–H)** Cytospectre graphs of cortical microtubule distribution corresponding to panel **(A)** in panel **(E)**, to panel **(B)** in panel **(F)**, to panel **(C)** in panel **(G),** and to panel **(D)** in panel **(H). (I–L)** Overview of hypocotyl of *max2-1* seedlings treated with solvent alone [mock; **(I)**], 3 μM of GR24 (**J**), 25 μM of GR24 **(K)**, and 3 μM of TIS108 **(L)**. **(M–P)** Cytospectre graphs of cortical microtubule distribution corresponding to panel **(I)** in panel **(M)**, to panel **(J)** in panel **(N)**, to panel **(K)** in panel **(O),** and to panel **(L)** in panel **(P)**. Scale bars: 20 μm.

Supplementary Figure 6 Assessment of microtubule organization in epidermal hypocotyl cells of dark-grown seedlings of Arabidopsis Col-0 wild type or the *max2-1* mutant expressing GFP-TUA6 construct in the presence or absence of GR24 synthetic strigolactone (3 μM and 25 μM) or the biosynthetic inhibitor of strigolactone production TIS108 (3 μM). **(A–D)** Overview of hypocotyl of Col-0 seedlings treated with solvent alone [mock; **(A)**], 3 μM of GR24 **(B)**, 25 μM of GR24 **(C)**, and 3 μM of TIS108 **(D)**. **(E–H)** Cytospectre graphs of cortical microtubule distribution corresponding to panel **(A)** in panel **(E)**, to panel **(B)** in panel **(F)**, to panel **(C)** in panel **(G),** and to panel **(D)** in panel **(H). (I–L)** Overview of hypocotyl of *max2-1* seedlings treated with solvent alone [mock; **(I)**], 3 μM of GR24 **(J)**, 25 μM of GR24 **(K)**, and 3 μM of TIS108 **(L)**. **(M–P)** Cytospectre graphs of cortical microtubule distribution corresponding to panel **(I)** in panel **(M)**, to panel **(J)** in panel **(N)**, to panel **(K)** in panel **(O),** and to panel **(L)** in panel **(P)**. Scale bars: 20 μm.

Supplementary Table 1 Statistical analysis for Figure 1Q.

Supplementary Table 2 Statistical analysis for Figure 1R.

Supplementary Table 3 Statistical analysis for Figure 2I.

Supplementary Table 4 Statistical analysis for Figure 3Q.

Supplementary Table 5 Statistical analysis for Figure 3R.

Supplementary Table 6 Statistical analysis for Figure 4R.

Supplementary Table 7 Statistical analysis for Figure 5I.

Supplementary Table 8 Statistical analysis for Figure 5K.

Supplementary Table 9 Statistical analysis for Figure 6K.

Supplementary Table 10 Statistical analysis for Figure 7Q.

Supplementary Table 11 Statistical analysis for Figure 7R.

Supplementary Table 12 Statistical analysis for Supplementary Figure 4A.

Supplementary Table 13 Statistical analysis for Supplementary Figure 4B.

Supplementary Movie 1 SIM time series corresponding to Figure 7A. Microtubule dynamics of etiolated mock-treated Col-0 hypocotyl epidermal cells expressing the GFP-MBD marker.

Supplementary Movie 2 SIM time series corresponding to Figure 7E. Microtubule dynamics of etiolated Col-0 hypocotyl epidermal cells expressing the GFP-MBD marker treated with 3 μM GR24.

Supplementary Movie 3 SIM time series corresponding to Figure 7I. Microtubule dynamics of etiolated Col-0 hypocotyl epidermal cells expressing the GFP-MBD marker treated with 25 μM GR24.

Supplementary Movie 4 SIM time series corresponding to Figure 7M. Microtubule dynamics of etiolated Col-0 hypocotyl epidermal cells expressing the GFP-MBD marker treated with 3 μM TIS108.

Supplementary Movie 5 SIM time series corresponding to Figure 8A. Microtubule dynamics of etiolated mock-treated *max2-1* hypocotyl epidermal cells expressing the GFP-MBD marker.

Supplementary Movie 6 SIM time series corresponding to Figure 8F. Microtubule dynamics of etiolated *max2-1* hypocotyl epidermal cells expressing the GFP-MBD marker treated with 3 μM GR24.

Supplementary Movie 7 SIM time series corresponding to Figure 8L. Microtubule dynamics of etiolated *max2-1* hypocotyl epidermal cells expressing the GFP-MBD marker treated with 25 μM GR24.

Supplementary Movie 8 SIM time series corresponding to Figure 8Q. Microtubule dynamics of etiolated *max2-1* hypocotyl epidermal cells expressing the GFP-MBD marker treated with 3 μM TIS108.
