# Supplementary material for: GR24, A Synthetic Strigolactone Analog, and Light Affect the Organization of Cortical Microtubules in Arabidopsis Hypocotyl Cells
Source: Front Plant Sci. 2021 Jul 7;12:675981. doi: 10.3389/fpls.2021.675981 (PMC8293678; doi:10.3389/fpls.2021.675981)
Supplement: Supplementary Figure 1 — Measurements of light-exposed (A) and etiolated (B) Col-0 and max2-1 hypocotyl length after treatment with GR24 or TIS108 (two-way ANOVA with Scheffé’s post hoc test; common letters denote groups without statistically significant differences at the 0.001 probability level; Supplementary Tables 1, 3). These measurements of hypocotyl growth were performed using a NeuronJ plugin for ImageJ (Meijering, 2010), as an alternative and complementary measuring approach. [file Presentation_1.pdf]

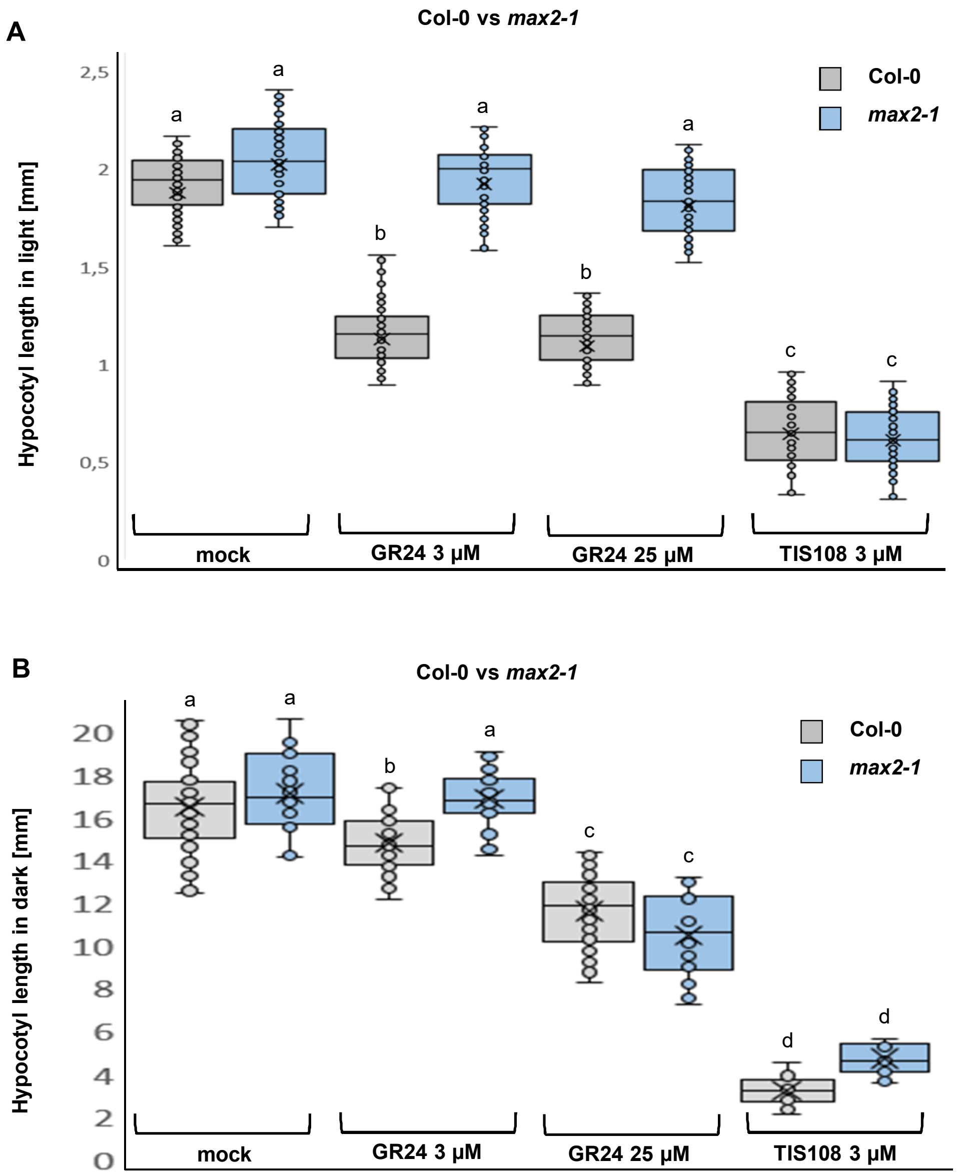


**Supplementary Figure 1.** Measurements of light-exposed **(A)** and etiolated **(B)** Col-0 and *max2-1* hypocotyl length after treatment with GR24 or TIS108 (two-way ANOVA with Scheffé's post-hoc test; common letters denote groups without statistically significant differences at the 0.001 probability level; **Supplementary Table 12**, **Supplementary Table 13**). These measurements of hypocotyl growth were performed using a NeuronJ plugin for ImageJ (Meijering, 2010).

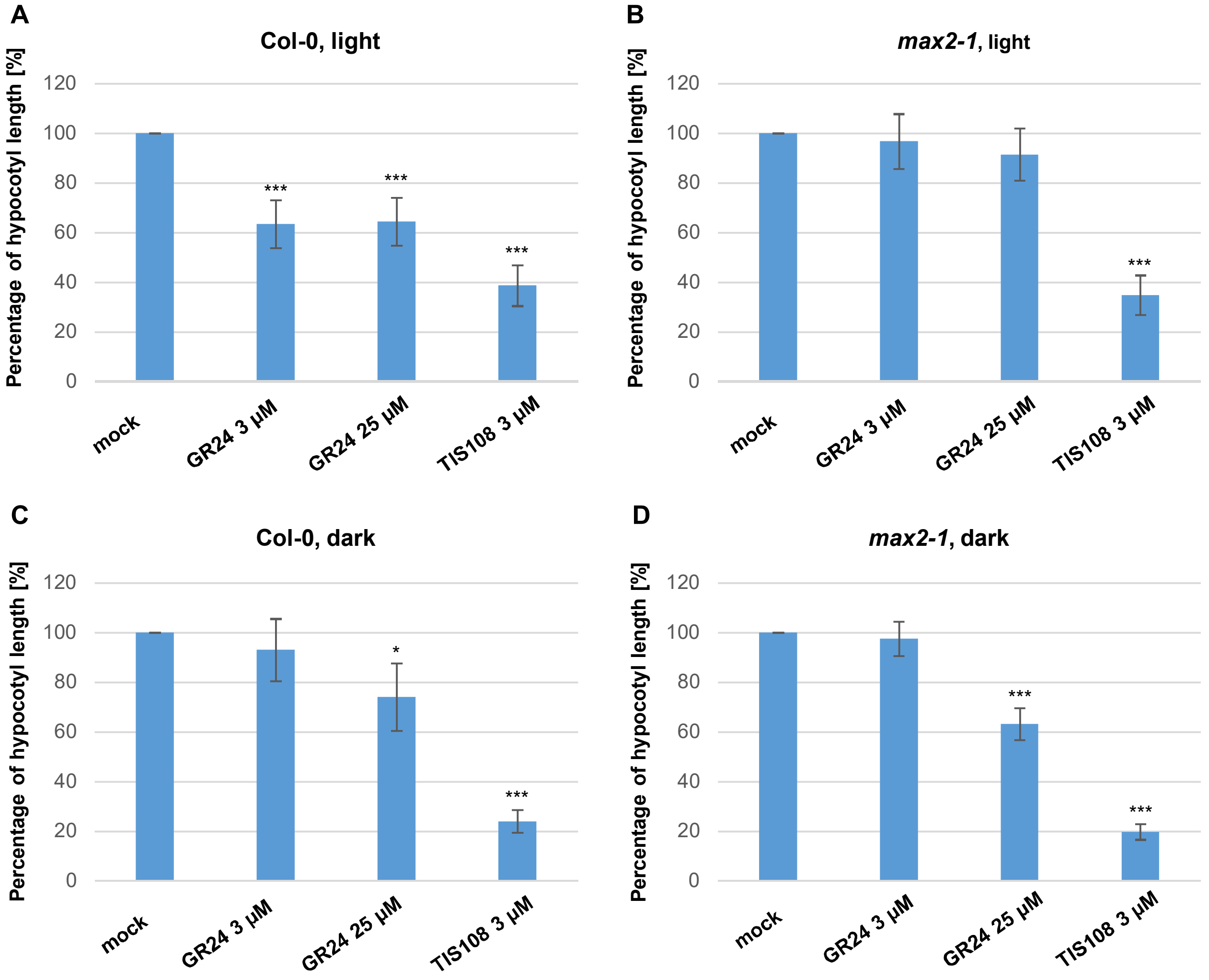


**Supplementary Figure 2.** Quantitative extent of the effect of GR24 interference in hypocotyl elongation of light-grown Col-0 **(A)**, light-grown *max2-1* **(B)**, and etiolated Col-0 **(C)** and *max2-1* **(D)** seedlings. *, $p<0.05$; ***, $p<0.001$ according to Student's t-test.

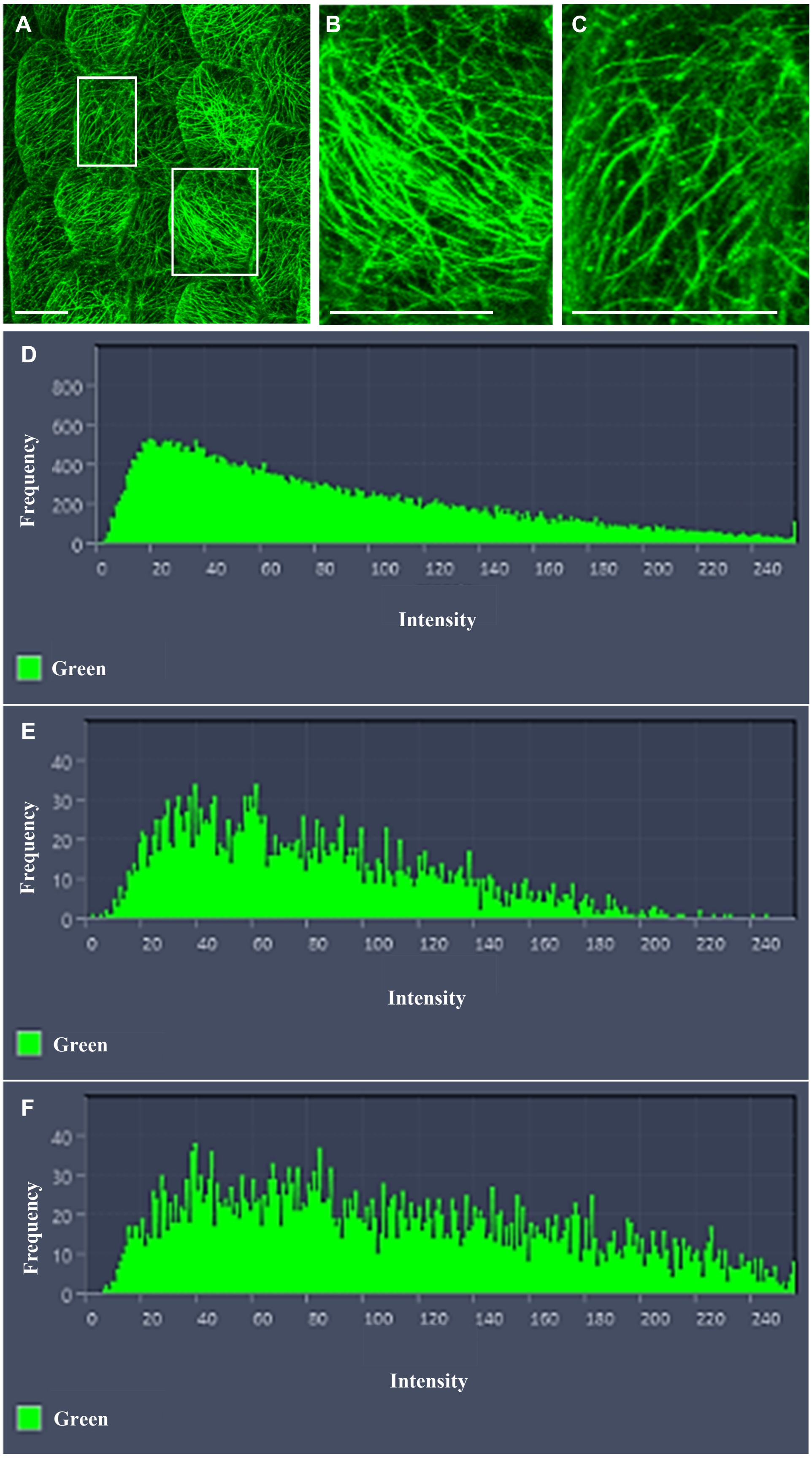


**Supplementary Figure 3.** Example of skewness determination as an indicator of microtubule bundling, based on **Figure 3B**. **(A)** Overview (same as **Figure 3B**). **(B)** Magnified view of the ROI corresponding to the bottom right boxed area of panel **(A)**. **(C)** Magnified view of the ROI corresponding to the top left boxed area of panel **(A)**. **(D-F)** Histograms of fluorescence intensity frequencies corresponding to panel (**A**) in panel **(D)**, to panel (**B**) in panel **(E)** and to panel (**C**) in panel **(F)**. Scale bars: 20 µm.

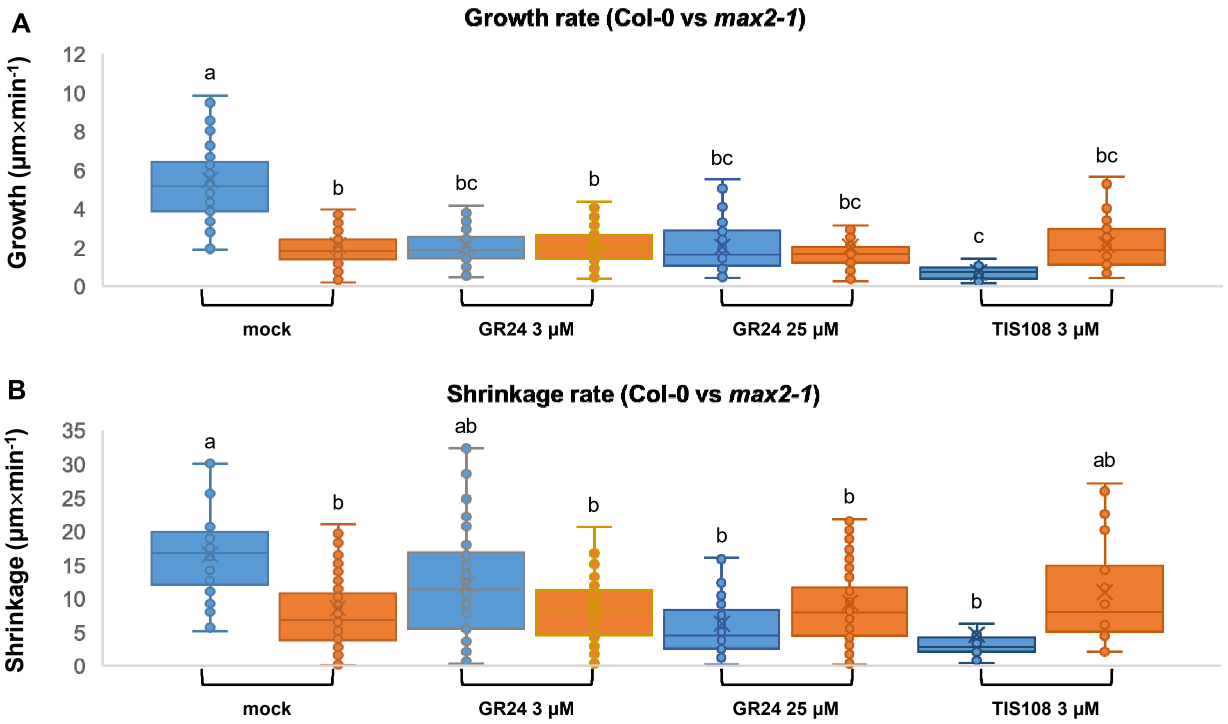


**Supplementary Figure 4.** Pairwise comparison of Col-0 wild type and *max2-1* microtubule plus end growth (**A**; (N≥33; two-way ANOVA was followed with Scheffé's test, statistical comparison is shown within groups sharing the same genotype; letters in the graph are shared by groups without statistically significant differences at the 0.001 probability level; results are in **Supplementary Table 12**) and shrinkage **(B**; N≥20; two-way ANOVA was followed with Scheffé's test, statistical comparison is shown within groups sharing the same genotype; letters in the graph are shared by groups without statistically significant differences at the 0.001 probability level; results are in **Supplementary Table 13**) under all experimental conditions used herein. In all box plots, average is presented by ×, median by the middle line, 1st quartile by the bottom line, 3rd quartile by the top line; the whiskers lie within the 1.5× interquartile range (defined from the 1st to the 3rd quartile) while outliers are omitted.

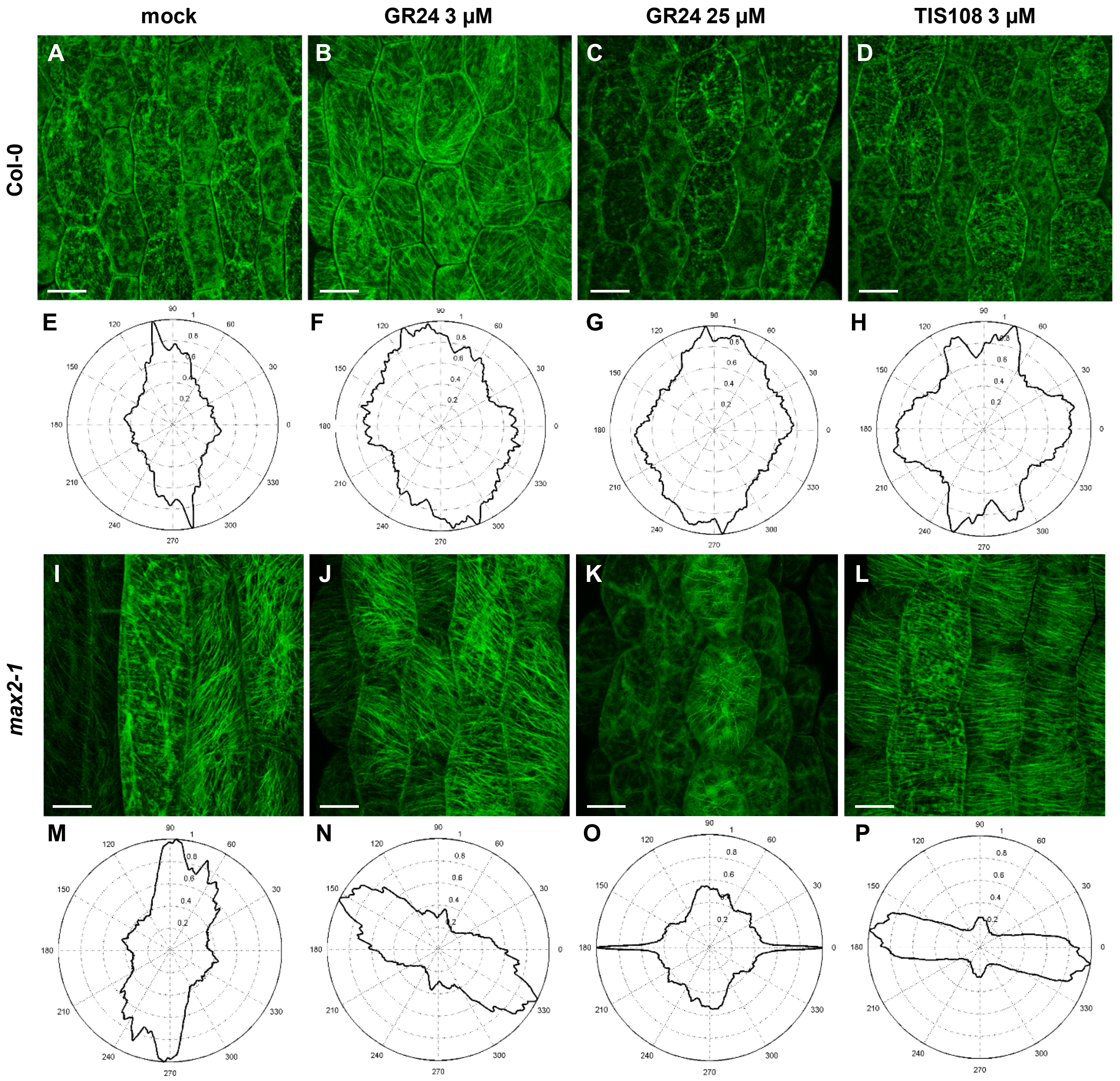


**Supplementary Figure 5.** Assessment of microtubule organization in epidermal hypocotyl cells of light-grown seedlings of Arabidopsis Col-0 wild type or the *max2-1* mutant expressing GFP-TUA6 construct in the presence or absence of GR24 synthetic strigolactone (3 µM and 25 µM) or the biosynthetic inhibitor of strigolactone production TIS108 (3 µM). **(A-D)** Overview of hypocotyl of Col-0 seedlings treated with solvent alone [mock; **(A)**], 3 µM of GR24 **(B)**, 25 µM of GR24 **(C)**, and 3 µM of TIS108 **(D)**. **(E-H)** Cytospectre graphs of cortical microtubule distribution corresponding to panel **(A)** in panel **(E)**, to panel **(B)** in panel **(F)**, to panel **(C)** in panel **(G)** and to panel **(D)** in panel **(H)**. **(I-L)** Overview of hypocotyl of *max2-1* seedlings treated with solvent alone [mock; **(I)**], 3 µM of GR24 (**J**), 25 µM of GR24 **(K)**, and 3 µM of TIS108 **(L)**. **(M-P)** Cytospectre graphs of cortical microtubule distribution corresponding to panel **(I)** in panel **(M)**, to panel **(J)** in panel **(N)**, to panel **(K)** in panel **(O)** and to panel **(L)** in panel **(P)**. Scale bars: 20 µm.

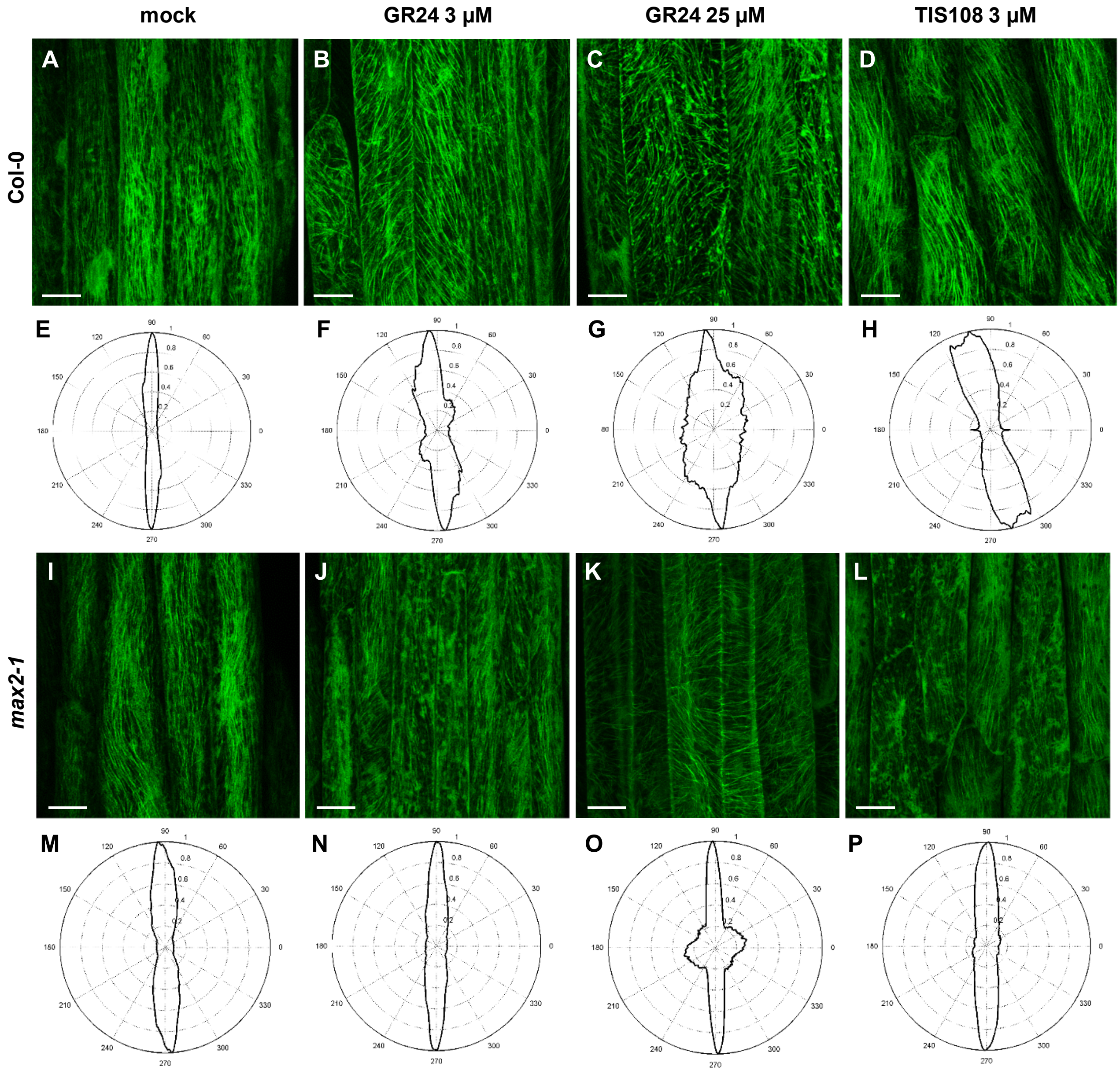


**Supplementary Figure 6.** Assessment of microtubule organization in epidermal hypocotyl cells of dark-grown seedlings of Arabidopsis Col-0 wild type or the *max2-1* mutant expressing GFP-TUA6 construct in the presence or absence of GR24 synthetic strigolactone (3 µM and 25 µM) or the biosynthetic inhibitor of strigolactone production TIS108 (3 µM). **(A-D)** Overview of hypocotyl of Col-0 seedlings treated with solvent alone [mock; **(A)**], 3 µM of GR24 **(B)**, 25 µM of GR24 **(C)**, and 3 µM of TIS108 **(D)**. **(E-H)** Cytospectre graphs of cortical microtubule distribution corresponding to panel **(A)** in panel **(E)**, to panel **(B)** in panel **(F)**, to panel **(C)** in panel **(G)** and to panel **(D)** in panel **(H)**. **(I-L)** Overview of hypocotyl of *max2-1* seedlings treated with solvent alone [mock; **(I)**], 3 µM of GR24 (**J**), 25 µM of GR24 **(K)**, and 3 µM of TIS108 **(L)**. **(M-P)** Cytospectre graphs of cortical microtubule distribution corresponding to panel **(I)** in panel **(M)**, to panel **(J)** in panel **(N)**, to panel **(K)** in panel **(O)** and to panel **(L)** in panel **(P)**. Scale bars: 20 µm.
