# Supplementary material for: GR24, A Synthetic Strigolactone Analog, and Light Affect the Organization of Cortical Microtubules in Arabidopsis Hypocotyl Cells
Source: Front Plant Sci. 2021 Jul 7;12:675981. doi: 10.3389/fpls.2021.675981 (PMC8293678; doi:10.3389/fpls.2021.675981)
Supplement: Supplementary Table 1 — Statistical analysis for Figure 1Q. [file Data_Sheet_1.docx]

**SUPPLEMENTARY TABLES**

**Supplementary Table 1***.* Statistical analysis for Fig. 1Q.

**Supplementary Table 2***.* Statistical analysis for Fig. 1R.

**Supplementary Table 3***.* Statistical analysis for Fig. 2I.

**Supplementary Table 4*.*** Statistical analysis for Fig. 3Q.

**Supplementary Table 5***.* Statistical analysis for Fig. 3R.

**Supplementary Table 6***.* Statistical analysis for Fig. 4R.

**Supplementary Table 7***.* Statistical analysis for Fig. 5I.

**Supplementary Table 8***.* Statistical analysis for Fig. 5K.

**Supplementary Table 9***.* Statistical analysis for Fig. 6K.

**Supplementary Table 10***.* Statistical analysis for Fig. 7Q.

**Supplementary Table 11***.* Statistical analysis for Fig. 7R.

**Supplementary Table 12***.* Statistical analysis for Suppl. Fig. 1A.

**Supplementary Table 13***.* Statistical analysis for Suppl. Fig. 1B.

**Supplementary Table 1.** Statistical analysis for Fig. 1Q.

| **two-way ANOVA followed by Scheffé's test; F (genotype; 1, 538)=669.4197, p=0.000; F (treatment; 3, 538)=1476.5402, p=0.000; F (3, 538)=189.1578, p=0.000** | | | | | | | | | |
| --- | --- | --- | --- | --- | --- | --- | --- | --- | --- |
| **p-values** | **genotype** | Col-0 | *max2* | Col-0 | *max2* | Col-0 | *max2* | Col-0 | *max2* |
| **genotype** | **treatment** | mock | mock | GR24 3 µM | GR24 3 µM | GR24 25 µM | GR24 25 µM | TIS 3 µM | TIS 3 µM |
| Col-0 | mock |  | 0.185 | 0 | 0.9999 | 0 | 0.2333 | 0 | 0 |
| *max2* | mock | 0.185 |  | 0 | 0.092 | 0 | 0 | 0 | 0 |
| Col-0 | GR24 3 µM | 0 | 0 |  | 0 | 0.9992 | 0 | 0 | 0 |
| *max2* | GR24 3 µM | 0.9999 | 0.092 | 0 |  | 0 | 0.0928 | 0 | 0 |
| Col-0 | GR24 25 µM | 0 | 0 | 0.9992 | 0 |  | 0 | 0 | 0 |
| *max2* | GR24 25 µM | 0.2333 | 0 | 0 | 0.0928 | 0 |  | 0 | 0 |
| Col-0 | TIS 3 µM | 0 | 0 | 0 | 0 | 0 | 0 |  | 0.9466 |
| *max2* | TIS 3 µM | 0 | 0 | 0 | 0 | 0 | 0 | 0.9466 |  |

**Supplementary Table 2.** Statistical analysis for Fig. 1R.

| **two-way ANOVA followed by Scheffé's test; F (genotype; 1, 407)=7.998, p=0.005; F (treatment; 3, 407)=8.930, p=0.000; F (3, 407)=2.816, p=0.039** | | | | | | | | | |
| --- | --- | --- | --- | --- | --- | --- | --- | --- | --- |
| **p-values** | **genotype** | Col-0 | *max2* | Col-0 | *max2* | Col-0 | *max2* | Col-0 | *max2* |
| **genotype** | **treatment** | mock | mock | GR24 3 µM | GR24 3 µM | GR24 25 µM | GR24 25 µM | TIS 3 µM | TIS 3 µM |
| Col-0 | mock |  | 0.0731 | 0.9712 | 0.0731 | 0.9541 | 0.9998 | 0.9987 | 1 |
| *max2* | mock | 0.0731 |  | 0.3978 | 1 | 0.0003 | 0.0504 | 0.6841 | 0.4123 |
| Col-0 | GR24 3 µM | 0.9712 | 0.3978 |  | 0.3978 | 0.2037 | 0.8745 | 1 | 0.9994 |
| *max2* | GR24 3 µM | 0.0731 | 1 | 0.3978 |  | 0.0003 | 0.0504 | 0.6841 | 0.4123 |
| Col-0 | GR24 25 µM | 0.9541 | 0.0003 | 0.2037 | 0.0003 |  | 0.9998 | 0.7951 | 0.9584 |
| *max2* | GR24 25 µM | 0.9998 | 0.0504 | 0.8745 | 0.0504 | 0.9998 |  | 0.9823 | 0.9991 |
| Col-0 | TIS 3 µM | 0.9987 | 0.6841 | 1 | 0.6841 | 0.7951 | 0.9823 |  | 1 |
| *max2* | TIS 3 µM | 1 | 0.4123 | 0.9994 | 0.4123 | 0.9584 | 0.9991 | 1 |  |

**Supplementary Table 3.** Statistical analysis for Fig. 2I.

| **two-way ANOVA followed by Scheffé's test; F (genotype; 1, 358)=11.19, p=0.001; F (treatment; 3, 358)=866.91, p=0.000; F (3, 358)=14.07, p=0.000** | | | | | | | | | |
| --- | --- | --- | --- | --- | --- | --- | --- | --- | --- |
| **p-values** | **genotype** | Col-0 | *max2* | Col-0 | *max2* | Col-0 | *max2* | Col-0 | *max2* |
| **genotype** | **treatment** | mock | mock | GR24 3 µM | GR24 3 µM | GR24 25 µM | GR24 25 µM | TIS 3 µM | TIS 3 µM |
| Col-0 | mock |  | 0.9442 | 0 | 0.9976 | 0 | 0 | 0 | 0 |
| *max2* | mock | 0.9442 |  | 0.0001 | 0.9998 | 0 | 0 | 0 | 0 |
| Col-0 | GR24 3 µM | 0 | 0.0001 |  | 0.0003 | 0 | 0 | 0 | 0 |
| *max2* | GR24 3 µM | 0.9976 | 0.9998 | 0.0003 |  | 0 | 0 | 0 | 0 |
| Col-0 | GR24 25 µM | 0 | 0 | 0 | 0 |  | 0.1077 | 0 | 0 |
| *max2* | GR24 25 µM | 0 | 0 | 0 | 0 | 0.1077 |  | 0 | 0 |
| Col-0 | TIS 3 µM | 0 | 0 | 0 | 0 | 0 | 0 |  | 0.0817 |
| *max2* | TIS 3 µM | 0 | 0 | 0 | 0 | 0 | 0 | 0.0817 |  |

**Supplementary Table 4.** Statistical analysis for Fig. 3Q.

| **Welch's ANOVA followed by Scheffé's test; F (3, 250)=6.2095, p=0.0006** | | | | |
| --- | --- | --- | --- | --- |
| **p-values for indicated treatment** | mock | GR24 3 µM | GR24 25 µM | TIS 3 µM |
| mock |  | 0.005 | 0.0375 | 0.2381 |
| GR24 3 µM | 0.005 |  | 0.9689 | 0.3957 |
| GR24 25 µM | 0.0375 | 0.9689 |  | 0.7528 |
| TIS 3 µM | 0.2381 | 0.3957 | 0.7528 |  |

**Supplementary Table 5.** Statistical analysis for Fig. 3R.

| **Welch's ANOVA followed by Scheffé's test; F (3, 202)=32.5002, p=0.0000** | | | | |
| --- | --- | --- | --- | --- |
| **p-values for indicated treatment** | mock | GR24 3 µM | GR24 25 µM | TIS 3 µM |
| mock |  | 0 | 0 | 0 |
| GR24 3 µM | 0 |  | 0.1232 | 0.0587 |
| GR24 25 µM | 0 | 0.1232 |  | 0.9933 |
| TIS 3 µM | 0 | 0.0587 | 0.9933 |  |

**Supplementary Table 6.** Statistical analysis for Fig. 4R.

| **Welch's ANOVA followed by Scheffé's test; F (3, 212)=3.31, p=0.0236** | | | | |
| --- | --- | --- | --- | --- |
| **p-values for indicated treatment** | mock | GR24 3 µM | GR24 25 µM | TIS 3 µM |
| mock |  | 0.0280 | 0.5612 | 0.1508 |
| GR24 3 µM | 0.0280 |  | 0.3532 | 0.6166 |
| GR24 25 µM | 0.5612 | 0.3532 |  | 0.9147 |
| TIS 3 µM | 0.1508 | 0.6166 | 0.9147 |  |

**Supplementary Table 7.** Statistical analysis for Fig. 5I.

| **Welch's ANOVA followed by Scheffé's test; F (3, 131)=67.0532, p=0.0000** | | | | |
| --- | --- | --- | --- | --- |
| **p-values for indicated treatment** | mock | GR24 3 µM | GR24 25 µM | TIS 3 µM |
| mock |  | 0 | 0 | 0 |
| GR24 3 µM | 0 |  | 0.9994 | 0.9437 |
| GR24 25 µM | 0 | 0.9994 |  | 0.9876 |
| TIS 3 µM | 0 | 0.9437 | 0.9876 |  |

**Supplementary Table 8.** Statistical analysis for Fig. 5K.

| **two-way ANOVA followed by Scheffé's test; F (genotype; 1, 292)=0.463, p=0.497; F (treatment; 3, 292)=54.468, p=0.000; F (3, 292)=52.118, p=0.000** | | | | | | | | | |
| --- | --- | --- | --- | --- | --- | --- | --- | --- | --- |
| **p-values** | **genotype** | Col-0 | *max2* | Col-0 | *max2* | Col-0 | *max2* | Col-0 | *max2* |
| **genotype** | **treatment** | mock | mock | GR24 3 µM | GR24 3 µM | GR24 25 µM | GR24 25 µM | TIS 3 µM | TIS 3 µM |
| Col-0 | mock |  | 0 | 0 | 0 | 0 | 0 | 0 | 0 |
| *max2* | mock | 0 |  | 0.2141 | 1 | 0.3843 | 1 | 0.0165 | 1 |
| Col-0 | GR24 3 µM | 0 | 0.2141 |  | 0.1722 | 1 | 0.3818 | 0.9996 | 0.1973 |
| *max2* | GR24 3 µM | 0 | 1 | 0.1722 |  | 0.3565 | 1 | 0.0076 | 1 |
| Col-0 | GR24 25 µM | 0 | 0.3843 | 1 | 0.3565 |  | 0.5513 | 1 | 0.3872 |
| *max2* | GR24 25 µM | 0 | 1 | 0.3818 | 1 | 0.5513 |  | 0.0526 | 1 |
| Col-0 | TIS 3 µM | 0 | 0.0165 | 0.9996 | 0.0076 | 1 | 0.0526 |  | 0.0099 |
| *max2* | TIS 3 µM | 0 | 1 | 0.1973 | 1 | 0.3872 | 1 | 0.0099 |  |

**Supplementary Table 9.** Statistical analysis for Fig. 6K.

| **two-way ANOVA followed by Scheffé's test; F (genotype; 1, 312)=23.058, p=0.000; F (treatment; 3, 312)=0.308, p=0.82; F (3, 312)=0.187, p=0.91** | | | | | | | | | |
| --- | --- | --- | --- | --- | --- | --- | --- | --- | --- |
| **p-values** | **genotype** | Col-0 | *max2* | Col-0 | *max2* | Col-0 | *max2* | Col-0 | *max2* |
| **genotype** | **treatment** | mock | mock | GR24 3 µM | GR24 3 µM | GR24 25 µM | GR24 25 µM | TIS 3 µM | TIS 3 µM |
| Col-0 | mock |  | 0.1901 | 0.9998 | 0.1077 | 0.9872 | 0.104 | 0.9992 | 0.0779 |
| *max2* | mock | 0.1901 |  | 0.4093 | 1 | 0.9294 | 1 | 0.838 | 1 |
| Col-0 | GR24 3 µM | 0.9998 | 0.4093 |  | 0.2861 | 0.9994 | 0.2695 | 1 | 0.2359 |
| *max2* | GR24 3 µM | 0.1077 | 1 | 0.2861 |  | 0.8997 | 1 | 0.7867 | 1 |
| Col-0 | GR24 25 µM | 0.9872 | 0.9294 | 0.9994 | 0.8997 |  | 0.8735 | 1 | 0.8932 |
| *max2* | GR24 25 µM | 0.104 | 1 | 0.2695 | 1 | 0.8735 |  | 0.752 | 1 |
| Col-0 | TIS 3 µM | 0.9992 | 0.838 | 1 | 0.7867 | 1 | 0.752 |  | 0.7732 |
| *max2* | TIS 3 µM | 0.0779 | 1 | 0.2359 | 1 | 0.8932 | 1 | 0.7732 |  |

**Supplementary Table 10.** Statistical analysis for Fig. 7Q.

| **Welch's ANOVA followed by Scheffé's test; F (3, 192)=83.122, p=0.0000** | | | | |
| --- | --- | --- | --- | --- |
| **p-values for indicated** treatment | mock | GR24 3 µM | GR24 25 µM | TIS 3 µM |
| mock |  | 0 | 0 | 0 |
| GR24 3 µM | 0 |  | 0.9996 | 0.0052 |
| GR24 25 µM | 0 | 0.9996 |  | 0.0050 |
| TIS 3 µM | 0 | 0.0052 | 0.0050 |  |

**Supplementary Table 11.** Statistical analysis for Fig. 7R.

| **Welch's ANOVA followed by Scheffé's test; F (3, 170)=33.524, p=0.0000** | | | | |
| --- | --- | --- | --- | --- |
| **p-values for indicated treatment** | mock | GR24 3 µM | GR24 25 µM | TIS 3 µM |
| mock |  | 0.0108 | 0 | 0 |
| GR24 3 µM | 0.0108 |  | 0.0009 | 0.0001 |
| GR24 25 µM | 0 | 0.0009 |  | 0.7912 |
| TIS 3 µM | 0 | 0.0001 | 0.7912 |  |

**Supplementary Table 12.** Statistical analysis for Suppl. Fig. 1A.

| **two-way ANOVA followed by Scheffé's test; F (genotype; 1, 793)=10.3368, p=0.001; F (treatment; 3, 793)=62.5420, p=0.000; F (3, 793)=64.6977, p=0.000** | | | | | | | | | |
| --- | --- | --- | --- | --- | --- | --- | --- | --- | --- |
| **p-values** | **genotype** | Col-0 | *max2* | Col-0 | *max2* | Col-0 | *max2* | Col-0 | *max2* |
| **genotype** | **treatment** | mock | mock | GR24 3 µM | GR24 3 µM | GR24 25 µM | GR24 25 µM | TIS 3 µM | TIS 3 µM |
| Col-0 | mock |  | 0 | 0 | 0 | 0 | 0 | 0 | 0 |
| *max2* | mock | 0 |  | 1 | 0.9956 | 1 | 1 | 0.0007 | 1 |
| Col-0 | GR24 3 µM | 0 | 1 |  | 0.9988 | 1 | 1 | 0.0227 | 1 |
| *max2* | GR24 3 µM | 0 | 0.9956 | 0.9988 |  | 0.9946 | 0.9744 | 0.0002 | 1 |
| Col-0 | GR24 25 µM | 0 | 1 | 1 | 0.9946 |  | 1 | 0.0217 | 1 |
| *max2* | GR24 25 µM | 0 | 1 | 1 | 0.9744 | 1 |  | 0.003 | 1 |
| Col-0 | TIS 3 µM | 0 | 0.0007 | 0.0227 | 0.0002 | 0.0217 | 0.003 |  | 0.0184 |
| *max2* | TIS 3 µM | 0 | 1 | 1 | 1 | 1 | 1 | 0.0184 |  |

**Supplementary Table 13.** Statistical analysis for Suppl. Fig. 1B.

| **two-way ANOVA followed by Scheffé's test; F (genotype; 1, 503)=0.2991, p=0.585; F (treatment; 3, 503)=11.8655, p=0.000; F (3, 503)=19.7909, p=0.000** | | | | | | | | | |
| --- | --- | --- | --- | --- | --- | --- | --- | --- | --- |
| **p-values** | **genotype** | Col-0 | *max2* | Col-0 | *max2* | Col-0 | *max2* | Col-0 | *max2* |
| **genotype** | **treatment** | mock | mock | GR24 3 µM | GR24 3 µM | GR24 25 µM | GR24 25 µM | TIS 3 µM | TIS 3 µM |
| Col-0 | mock |  | 0 | 0.1732 | 0 | 0 | 0 | 0 | 0.0779 |
| *max2* | mock | 0 |  | 0.2369 | 0.9999 | 0.8527 | 0.9972 | 0.4079 | 1 |
| Col-0 | GR24 3 µM | 0.1732 | 0.2369 |  | 0.5679 | 0.0307 | 0.6941 | 0.0056 | 0.2359 |
| *max2* | GR24 3 µM | 0 | 0.9999 | 0.5679 |  | 0.7144 | 1 | 0.2862 | 1 |
| Col-0 | GR24 25 µM | 0 | 0.8527 | 0.0307 | 0.7144 |  | 0.5736 | 0.9958 | 0.8932 |
| *max2* | GR24 25 µM | 0 | 0.9972 | 0.6941 | 1 | 0.5736 |  | 0.1938 | 1 |
| Col-0 | TIS 3 µM | 0 | 0.4079 | 0.0056 | 0.2862 | 0.9958 | 0.1938 |  | 0.7732 |
| *max2* | TIS 3 µM | 0.231 | 0.96 | 0.9997 | 0.993 | 0.5502 | 0.9978 | 0.2293 |  |
